# A new method of metabarcoding Microsporidia and their hosts reveals high levels of microsporidian infections in mosquitoes (Culicidae)

**DOI:** 10.1111/1755-0998.13205

**Published:** 2020-07-01

**Authors:** Artur Trzebny, Anna Slodkowicz‐Kowalska, James J. Becnel, Neil Sanscrainte, Miroslawa Dabert

**Affiliations:** ^1^ Molecular Biology Techniques Laboratory Faculty of Biology Adam Mickiewicz University Poznan Poland; ^2^ Department of Biology and Medical Parasitology Faculty of Medicine I University of Medical Sciences Poznan Poland; ^3^ USDA Agricultural Research Service Center for Medical, Agricultural and Veterinary Entomology Gainesville FL USA

**Keywords:** co‐infection, DNA barcoding, molecular diagnostics, molecular phylogeny, *Nosema* spp., rDNA

## Abstract

DNA metabarcoding offers new perspectives, especially with regard to the high‐throughput identification and diagnostics of pathogens. Microsporidia are an example of widely distributed, opportunistic and pathogenic microorganisms in which molecular identification is important for both environmental research and clinical diagnostics. We have developed a method for parallel detection of both microsporidian infection and the host species. We designed new primer sets: one specific for the classical Microsporidia (targeting the hypervariable V5 region of small subunit [ssu] rDNA), and a second one targeting a shortened fragment of the *COI* gene (standard metazoan DNA‐barcode); both markers are well suited for next generation sequencing. Analysis of the ssu rDNA data set representing 607 microsporidian species (120 genera) indicated that the V5 region enables identification of >98% species in the data set (596/607). To test the method, we used microsporidians that infect mosquitoes in natural populations. Using mini‐COI data, all field‐collected mosquitoes were unambiguously assigned to seven species; among them almost 60% of specimens were positive for at least 11 different microsporidian species, including a new microsporidian ssu rDNA sequence (Microsporidium sp. PL01). Phylogenetic analysis showed that this species belongs to one of the two main clades in the Terresporidia. We found a high rate of microsporidian co‐infections (9.4%). The numbers of sequence reads for the operational taxonomic units suggest that the occurrence of *Nosema* spp. in co‐infections could benefit them; however, this observation should be retested using a more intensive host sampling. Our results show that DNA barcoding is a rapid and cost‐effective method for deciphering sample diversity in greater resolution, including the hidden biodiversity that may be overlooked using classical methodology.

## 1 INTRODUCTION

Taberlet, Coissac, Pompanon, Brochmann, and Willerslev (2012) proposed next‐generation biodiversity assessment using DNA metabarcoding. Since then automated identification of multiple species from a single bulk sample has become more accessible through increased availability of next‐generation sequencing (NGS) platforms and bioinformatic tools for NGS data analysis (e.g., Krehenwinkel, Pomerantz, & Prost, 2019; Ruppert, Kline, & Rahman, 2019; Zepeda Mendoza, Sicheritz‐Pontén, & Thomas Gilbert, 2015). DNA metabarcoding, which allows for rapid and cost‐effective identification of the biodiversity of the entire sample across a wide range of taxa and habitats, has proved its usefulness for prokaryotic and eukaryotic species identification. Many examples of this approach have been provided in biodiversity assessment of bacteria (e.g., Adamczyk et al., 2019; Cordier, 2019; Thapa, Zhang, & Allen, 2019), fungi (e.g., Hu et al., 2019; Luis et al., 2019; Xiao et al., 2019), plants (e.g., Bell et al., 2019; Tnah et al., 2019; Veldman et al., 2020) and animals from micro‐ to megafauna (e.g., Dopheide et al., 2019; Head et al., 2018; Lynggaard et al., 2019). However, there are still groups of organisms for which metabarcoding could offer new perspectives, especially with regard to high‐throughput identification and diagnostics of pathogens and pests (Tedersoo, Drenkhan, Anslan, Morales‐Rodriguez, & Cleary, 2018). Furthermore, NGS‐based methods are increasingly being recognized as pivotal for both the detection and the identification of pathogens, especially in cases of emerging infectious diseases, and in pathogens with complex life histories and co‐infections (Hernández‐Andrade et al., 2019; Näpflin et al., 2019).

Members of the phylum Microsporidia are an example of widely distributed opportunistic and pathogenic microorganisms for which molecular identification is important for both environmental research and clinical diagnostics. Microsporidians are a large and diverse group of obligate intracellular eukaryotic parasites that infect nearly every animal phylum, including other parasites, and certain protist species. The microsporidian spores are the only developmental stage with the ability to survive outside the host cell (Franzen, 2004). Infectious spores accumulate in the environment, when they are released in faeces or when an infected host dies (Campbell, van Frankenhuyzen, & Smith, 2007; Goertz & Hoch, 2008; Hoch, D’Amico, Solter, Zubrik, & McManus, 2008).

The most common way in which microsporidians spread is the horizontal transmission of infectious spores from food or the environment (Didier & Weiss, 2011). Microsporidian spores have been detected in drinking and wastewater (e.g., Ayed et al., 2012; Galván et al., 2013; Li et al., 2012) as well as in irrigation and surface water, including in recreational areas (e.g., Coupe et al., 2006; Graczyk, Sunderland, Tamang, Lucy, & Breysse, 2007; Lucy, Graczyk, Tamang, Miraflor, & Minchin, 2008). Using vertical transmission, microsporidians mostly infect ovaries and associated reproductive structures of the host (e.g., Becnel, Hazard, Fukuda, & Sprague, 1987; Becnel, Sprague, Fukuda, & Hazard, 1989; Goertz, Solter, & Linde, 2007; Kellen & Lindegren, 1973). Other transmission routes include surface‐contaminated host eggs (e.g., Alger & Undeen, 1970; Vávra & Undeen, 1970) or require obligatory development in an intermediate host (e.g., Andreadis, 2002; Micieli, García, & Andreadis, 2009; Sweeney, Doggett, & Piper, 1993).

The phylum Microsporidia consists of three evolutionary lineages: the so‐called "classical Microsporidia" and two poorly studied groups, the Metchnikovellida and the Chytridiopsida. This classification is based on structural and developmental characteristics (Vossbrinck, Debrunner‐Vossbrinck, & Weiss, 2014) and has been supported by molecular phylogenetic analyses (Bass et al., 2018; Corsaro et al., 2019). Classical Microsporidia are the largest group in the phylum, with more than 1,500 described species belonging to at least 200 genera (Becnel, Takvorian, & Cali, 2014; Vávra & Lukeš, 2013). Moreover, this group includes 17 species causing human disease; the most common belong to the genera *Enterocytozoon* and *Encephalitozoon* (Fayer & Santin‐Duran, 2014; Franzen & Müller, 2001; Stentiford et al., 2016). In immunosuppressed hosts, including humans, Microsporidia can infect any organ system; the most widely reported infections concern encephalitis, myositis, ocular infection and sinusitis (Sharma, Balne, & Das, 2014; Wang et al., 2018; Weiss, 2014; Weiss & Schwartz, 2015).

The real species diversity in this group is probably largely unknown, as Microsporidia are generally studied as zoonotic and/or waterborne agents of human disease or veterinary parasites. Sometimes, microsporidians are recorded incidentally during a fine structure analysis of their hosts (Radek, Kariton, Dabert, & Alberti, 2015; Ribeiro & Passos, 2006). Many microsporidian species are known only based on unique DNA sequence coding for nuclear ribosomal RNA (hereafter: rDNA; Krebes, Blank, Frankowski, & Bastrop, 2010; Vossbrinck, Andreadis, Vávra, & Becnel, 2004; Williams, Hamilton, Jones, & Bass, 2018).

Difficulties in the identification of microsporidians result mainly from their specific morphological and genomic characteristics. The classical microsporidian cell is characterized by mitochondria significantly reduced to mitosomes (Vávra, 2005; Williams, Hirt, Lucocq, & Embley, 2002), the absence of Golgi apparatus (Beznoussenko et al., 2007; Vávra & Lukeš, 2013) and the lack of peroxisomes or other simple organelles of this type (Fast, Law, Williams, & Keeling, 2003; Vávra & Lukeš, 2013). In addition, their ribosomes have a prokaryote‐like structure with subunits of 50S and 30S (Ishihara & Hayashi, 1968). Currently, the length and bending angle of the polar filament along with life cycle observations are the main morphological diagnostic features for microsporidian species identification (Xu & Weiss, 2005). Increasingly, the description of new species is being supplemented by molecular data (Nishikori et al., 2018; Sokolova & Overstreet, 2018; Vávra, Fiala, Krylová, Petrusek, & Hyliš, 2019).

Standard Microsporidia detection methods are based on ultrastructural assessment of an infected material. Light microscopy‐based methods mainly consist of the detection of a thick chitin wall of spores using different stains (Field, Hing, Milliken, & Marriott, 1993; Ignatius et al., 1997; Moura et al., 1997; Peterson, Spitsbergen, Feist, & Kent, 2011; van Gool et al., 1993). Staining for light microscopy rarely enables species identification, and electron microscopy has low sensitivity because only a small amount of sample can be examined (Weber, Bryan, Schwartz, & Owen, 1994). To identify pathogenic microsporidia in clinical samples, antigen‐based detection assays are used to recognize characteristic pathogen‐specific antigens, mostly located in the spore wall or the polar tube (del Aguila et al., 1998; Furuya et al., 2008; Luján et al., 1998; Singh, Sheoran, Zhang, Carville, & Tzipori, 2005; Zhang et al., 2005). Serological tests are rarely used because they can lead to false results (van Gool et al., 1997; Hollister, Canning, & Willcox, 1991; Kučerová‐Pospišilová & Ditrich, 1998).

Nucleic acid‐based detection methods are used for both clinical and environmental applications. The methods used in medical diagnostics are strictly targeted. Usually, they involve nested‐ or quantitative‐PCR techniques employing species‐specific primers (Ghosh, Schwartz, & Weiss, 2014; Ghosh & Weiss, 2009; Ghoyounchi et al., 2019; Javanmard et al., 2018) and probes (Huibers et al., 2018; Menotti et al., 2003; Verweij, Ten Hove, Brienen, & van Lieshout, 2007; Wang, Orlandi, & Stenger, 2005; Wolk, Schneider, Wengenack, Sloan, & Rosenblatt, 2002). Microsporidia detection in natural populations, by contrast, is based on end‐point PCR amplification of rDNA fragments and direct Sanger sequencing of the resulting amplicons. Most often, as a target for sequencing, researchers amplify fragments of the nuclear small subunit rRNA gene (hereafter: ssu rDNA) using Microsporidia‐specific (Weiss & Vossbrinck, 1998) or semispecific primer sets (e.g., Emsen et al., 2016; Grabner, 2017; Grabner et al., 2015; Quiles et al., 2019). Presently, the primer pair V1F (Zhu et al., 1993) and 530R (Baker, Vossbrinck, Didier, Maddox, & Shadduck, 1995) seems to be the most commonly used for the detection of microsporidian DNA. The V1F/530R primer set targets a fragment of about 400 bp which covers hypervariable V1–V3 regions of ssu rDNA. This approach has been shown to be effective in numerous microsporidian lineages (e.g., Bojko et al., 2015; Evans, Llanos, Kunin, & Evison, 2018; Simakova, Tokarev, & Issi, 2018; Sokolova & Overstreet, 2018; Wattier et al., 2007). However, in the case of co‐infection with different microsporidian species, such an approach based on direct amplicon sequencing using a conventional method could fail due to ambiguous Sanger sequencing results or can yield false results if one of the species dominates in the sample.

The first attempt to use high‐throughput sequencing for microsporidian DNA detection was performed by Williams et al. (2018). They applied the standard V1F and 530R primers to check the diversity of Microsporidia in environmental samples. Using this primer set, they were able to uncover a previously unknown microsporidian diversity; however, their raw data contained mostly nontarget sequences (see figure 2 in Williams et al., 2018). The high percentage of nonmicrosporidian sequences found in their study suggests that the standard V1F/530R primer set is not specific enough to amplify exclusively microsporidian DNA.

The present study aimed to develop a new molecular method for the rapid and sensitive detection of microsporidia using a DNA marker better suited for an NGS approach. Additionally, we present a new primer set to amplify the short DNA barcode based on the mitochondrial cytochrome *c* oxidase subunit I (*COI*) gene for parallel identification of the microsporidian host species. As a model, we used microsporidia that infect mosquitoes (Culicidae). Microsporidia are common in mosquitoes: over 90 microsporidian species have been recorded worldwide from this host. In addition, some microsporidians parasitic in mosquitoes also infect other species of insects, crustaceans and vertebrates (Andreadis, 2007; Becnel, White, & Shapiro, 2005; Vossbrinck et al., 2004).

## 2 MATERIAL AND METHODS

### 2.1 Material

To determine the sensitivity of the metabarcoding method we used three commercial lines of microsporidian spores: *Encephalitozoon cuniculi* (P103C, 1 × 10^6^), *E. hellem* (P103H, 1 × 10^6^) and *E. intestinalis* (P103I, 1 × 10^6^) from Waterborne Inc. For each species, a series of 10‐fold dilutions were prepared, ranging from 10 to 10,000 spores/ml. In addition, to prove that the new method is able to recover microsporidian species in co‐infections, four samples consisting of a mixture of *E. cuniculi*, *E. hellem* and *E. intestinalis* spores were prepared, ranging from 10:10:10 to 10:10:10,000 spores, respectively.

As positive controls, we used DNA isolates of *Anncaliia algerae* and *Vavraia culicis* from the Mosquito and Fly Research Unit, USDA‐ARS Center for Medical, Agricultural and Veterinary Entomology (CMAVE), Gainesville, FL, USA, and a DNA sample of *Enterocytozoon bieneusi* isolated in the Department of Biology and Medical Parasitology, Faculty of Medicine I, University of Medical Sciences, Poznan, Poland.

As microsporidia‐negative controls, we used colony mosquitoes believed to be free of microsporidia bred at the USDA‐ARS‐CMAVE. In total, we analysed 200 mosquito individuals representing four species of 50 individuals each: *Aedes aegypti*, *Anopheles quadrimaculatus*, *Culex quinquefasciatus* and *Uranotaenia lowii*.

Field‐collected mosquito samples consisted of 212 adult females collected between July and August 2016 from the periphery of mixed birch–oak and riparian forests, near the city of Poznan, western Poland. Mosquitoes were collected at night using human‐landing catches. Captured mosquitoes were preserved in 80% ethanol at 4°C until DNA extraction.

### 2.2 DNA extraction

Before DNA extraction, mosquitoes were washed in 96% ethyl alcohol (washing solution). Individual mosquitoes were placed in 1.5‐ml Eppendorf tubes until DNA extraction, while the washing solution was filtered through the MF‐Millipore Membrane Filter, 0.22 µm pore size (Merck). The filter was then cut and placed in an Eppendorf tube containing 180 µl ATL lysis buffer (Qiagen) and incubated with 0.2 mg of Proteinase K (Bio Basic) for 48 hr at 56°C. Later, 100 µl of the lysate was used for column‐based DNA extraction using the DNeasy Blood & Tissue Kit (Qiagen) according to the manufacturer's protocol for animal tissues.

Genomic DNA was isolated from spores and mosquitoes using a modified ammonium hydroxide method (Rijpkema & Bruinink, 1996). Two hundred microlitres of 0.7 M ammonium hydroxide (POCH S.A.) was added to 100 µl of the spore suspension or to one mosquito individual and homogenized for 30 s using the Pellet Cordless Motor Pellet (DWK Life Sciences) with disposable micro pestles (Scientific Specialties Inc.). Samples were incubated for 20 min at 99°C with shaking and the tubes were then opened and further left under the same conditions to concentrate the lysate to about 100µl volumes. The samples were then centrifuged for 5 min at 14,100 x g, and the supernatant was collected. Before PCR amplification, DNA extracts from mosquitoes were normalized with sterile water to a concentration of ~10 ng/µl.

### 2.3 Standard molecular approach for microsporidia detection

We compared the performance of our new metabarcoding approach with the standard PCR‐based detection method. We amplified and sequenced the V1–V3 region of ssu rDNA using primers V1F (CACCAGGTTGATTCTGCCTGAC) and 530R (CCGCGGCKGCTGGCAC). PCRs were prepared in two technical replicates, each in a total volume of 10 µl containing Hot FIREPol DNA Polymerase (Solis BioDyne), 0.25 µm of each primer and 1 µl of template DNA. The amplification programme was as follows: 12 min at 95°C, followed by 35 cycles of 15 s at 95°C, 1 min at 60°C and 1 min at 72°C, with a final extension step at 72°C for 10 min. DNA isolated from 100 spores of *E. intestinalis* was used as a positive control. After amplification, technical replications were pooled.

Standard *COI*‐barcode (Hebert, Cywinska, Ball, & deWaard, 2003) was amplified using primers bcdF01 and bcdR04 (Dabert, Witalinski, Kazmierski, Olszanowski, & Dabert, 2010). PCRs were prepared in a volume of 10 µl containing Hot FIREPol DNA Polymerase, 0.5 µm of each primer and 1 µl of template DNA. The PCR programme was as follows: 12 min at 95°C, followed by 35 cycles of 15 s at 95°C, 30 s at 50°C and 1 min at 72°C, with a final extension step at 72°C for 5 min. DNA isolated from *Aedes aegypti* was used as a positive control.

Five microlitres of the PCR was analysed by electrophoresis on a 1.5% agarose gel stained with GelRed (Biotium) according to the manufacturer's instruction. Samples containing visible bands were purified with *Escherichia coli* exonuclease I and FastAP Alkaline Phosphatase (Thermo Scientific) and sequenced using a BigDye version 3.1 kit and ABI Prism 3130XL Genetic Analyzer (Applied Biosystems), following the manufacturer's instructions. Sequence chromatograms were checked for accuracy and, if necessary, manually edited in geneious R11.1.5 (Biomatters).

### 2.4 Designing new primers for microsporidia detection

A new microsporidia‐specific primer set was developed based on ssu rDNA sequence data published in GenBank. In total, 1,133 sequences representing 120 genera belonging to the classical Microsporidia were aligned using mafft and L‐INS‐i algorithm (Katoh, Misawa, Kuma, & Miyata, 2002; Katoh & Standley, 2013) as implemented in geneious R11.1.5. Primers were manually designed to cover an ~200‐bp fragment coding for the helices 27–34 in the *Heterosporis anguillarum* ssu rDNA secondary structure (Tsai, Kou, Lo, & Wang, 2002); according to Neefs, Van de Peer, De Rijk, Chapelle, and de Wachter (1993) this region covers the V5 hypervariable region of ssu rRNA (V5 region). The primer sequences were analysed in oligo analyzer version 3.1 (Integrated DNA Technologies) to check the difference of melting temperatures and to search for possible primer secondary structures.

The percentage identities of the aligned V1–V3 and V5 regions were estimated using the Kolmogorov–Smirnov statistical test in the genedoc version 2.7 sequence editing tool (Nicholas & McClain, 1995). The final data set for V1–V3 and V5 alignments used in the analysis consisted of 649 and 607 available microsporidian sequences, respectively. Duplicate reads were extracted from the data set using the default settings in Dᴇᴅᴜᴘᴇ Dᴜᴘʟɪᴄᴀᴛᴇ Rᴇᴀᴅ Rᴇᴍᴏᴠᴇʀ version 37.64 implemented in geneious R11.1.5.

### 2.5 Amplification of the V5 region and mini‐COI for NGS sequencing

The microsporidian V5 region was amplified using primers CM‐V5F (GATTAGANACCNNNGTAGTTC) and CM‐V5R (TAANCAGCACAMTCCACTC) developed in this study. Mosquito species were determined by DNA‐barcoding using the shortened (373‐bp) fragment of the mitochondrial *COI* gene (hereafter: mini‐COI) covering 5' fragment of the standard DNA‐barcode. The mini‐COI was amplified using a primer pair developed in this study: bcdF01 (CATTTTCHACTAAYCATAARGATATTGG) (Dabert et al., 2010) and bcdR06 (GGDGGRTAHACAGTYCAHCCNGT). All PCR primers for NGS sequencing used in this study were tailed at 5' ends with dual‐indexed Ion Torrent adapters (forward tail 5'‐CCATCTCATCCCTGCGTGTCTCCGACTCAG‐index‐GAT, reverse tail 5'‐CCTCTCTATGGGCAGTCGGTGAT‐index) for sequencing using the Ion Torrent system (Life Technologies).

The V5 region was amplified in two technical replications, each in a total volume of 10 µl containing Hot FIREPol DNA Polymerase, 0.25 µm of each tailed primer and 1 µl of template DNA. The PCR programme was as follows: 12 min at 95°C, followed by 40 cycles of 15 s at 95°C, 30 s at 50°C and 30 s at 72°C, with a final extension step at 72°C for 5 min.

PCR amplification of mini‐COI was performed in a volume of 5 µl containing Hot FIREPol DNA Polymerase, 0.25 µm of each tailed primer and 1 µl of template DNA. The PCR programme was as follows: 12 min at 95°C, followed by 35 cycles of 15 s at 95°C, 30 s at 50°C and 45 s at 72°C, with a final extension step at 72°C for 5 min.

### 2.6 Library construction and NGS sequencing

**Figure 1:**
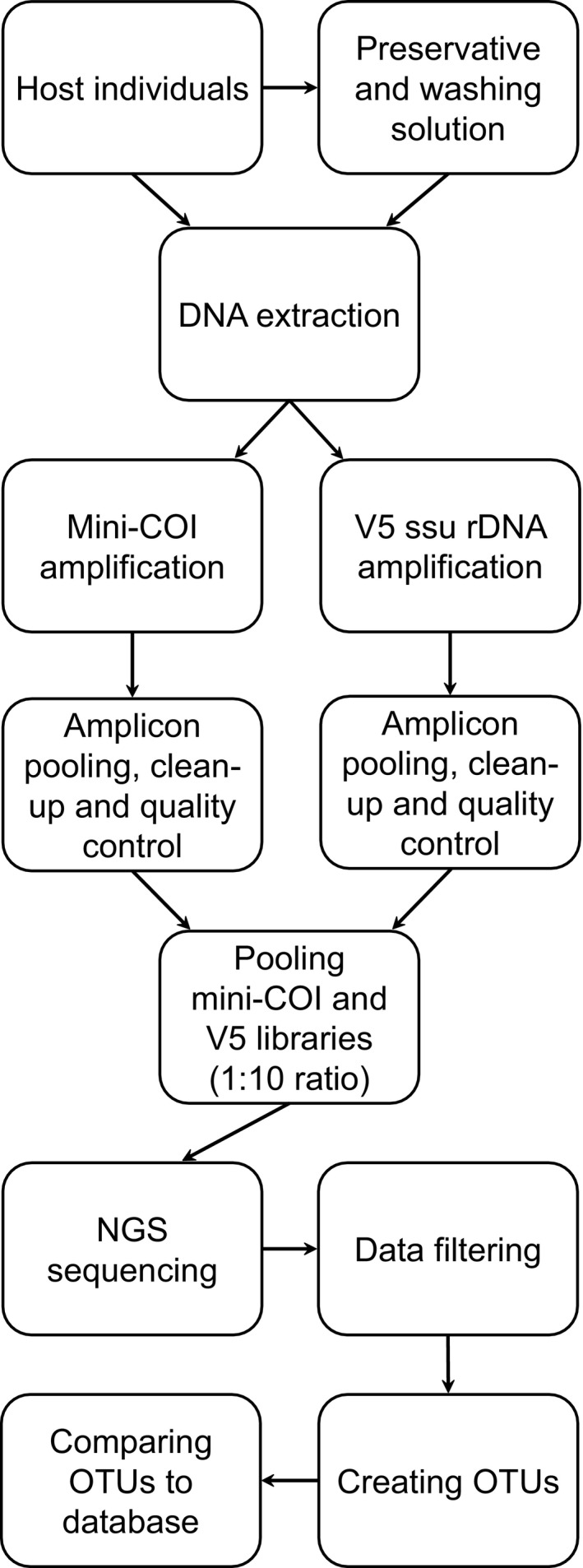
Protocol for metabarcoding of microsporidia and their hosts. Total genomic DNA is extracted from each previously washed host specimen and from the medium used for host preservation and washing. The mini‐COI barcode and the V5 region of ssu rDNA are amplified using PCR primers fused with double‐indexed NGS adaptors. The mini‐COI and V5 libraries are prepared separately, and after quality control, pooled in a ratio of 1:10, respectively. The libraries are NGS‐sequenced using a threshold of at least 10,000 reads per sample. After data quality filtering, the OTUs are clustered, and then compared to databases with reference sequences

The V5 region and mini‐COI libraries were prepared separately (Figure 1). For each PCR, 3 µl was electrophoresed on a 2% agarose gel to check amplification efficiency. The V5 region amplicons were pooled based on DNA band intensities. The sample volumes ranged from 5 µl, where the amplicons were invisible on the gel, to 1 µl in the case of brighter bands. The rare samples which had very high intensity were diluted 100‐fold with sterile water before pooling. For the mini‐COI library, all samples were pooled using 1 µl of each PCR. The V5 region and mini‐COI libraries were purified separately using a 2% E‐Gel SizeSelect II Agarose Gels system (Invitrogen), according to the manufacturer's instructions. DNA concentration and fragment length distribution of the libraries were established with the use of a High Sensitivity D1000 Screen Tape assay on 2200 Tape Station system (Life Technologies).

Sequence data used in this study were generated in several independent sequencing experiments. Clonal template amplifications were performed using the Ion Torrent One Touch System II and the Ion Torrent OT2 Kit according to the manufacturer's instructions. For the emulsion PCR, the V5 region and mini‐COI libraries were pooled in a 10:1 ratio. Sequencing was carried out using a Hi‐Q View Sequencing Kit and Ion PGM system on Ion 314 and Ion 318 chips or Ion 520 & Ion 530 Kit‐OT2 and S5 system on Ion 530 chips (Life Technologies) according to the manufacturer's instructions. Samples were pooled for each sequencing to obtain at least 100,000 reads per sample. Negative controls from blank DNA extractions and PCR reagents were included in each PCR and sequencing experiment.

### 2.7 Read processing and data analysis

Raw sequence data were pre‐filtered by Iᴏɴ Tᴏʀʀᴇɴᴛ Sᴜɪᴛᴇ software version 5.10.1 (Life Technologies) to remove polyclonal and low‐quality sequences. Further bioinformatic analysis was conducted using a fastq data and custom workflow. Sequence reads shorter than180 bp were removed from the data set. Leading and trailing low‐quality bases or Ns were removed using trimmomatic version 0.39 (Bolger, Lohse, & Usadel, 2014). The fastx toolkit (Hannon, 2010) was used to extract sequences with a minimum of 50% of bases with a quality score ≥ 25. Quality filtered sequences were separated into individual combinations of indexes in geneious R11.1.5. Chimeras were removed using the default settings in uchime2 version 4.2.40 (Edgar, 2016) and SILVA database for ARB for SSU rRNAs version 132 (Glöckner et al., 2017; Quast et al., 2013; Yilmaz et al., 2014) as implemented in geneious R11.1.5. Next, the sequences were trimmed at the 5’ and 3’ ends to exclude PCR primers.

Operational taxonomic unit (OTU) clustering was done in usearch version 11.0.667 (Edgar, 2010). Singletons (<10 reads) were removed, then OTUs were clustered from the sequences whose abundance exceeded a threshold of 10 counts using the cluster_otus algorithm (Edgar, 2013). The OTU consensus sequences were compared to GenBank using blastn (Zhang, Schwartz, Wagner, & Miller, 2000) optimized for highly similar sequences (megablast algorithm) (Morgulis et al., 2008).

We used a 97% identity threshold to determine mosquito species, and 100% identities for the identification of microsporidian species. The sequences generated are available in GenBank under accession nos. MT001301–MT001427, MT015707–MT015901 and MT075548–MT075550 (Table S1).

### 2.8 Phylogenetic analyses

To confirm the taxonomic position of the Microsporidia detected in field‐collected mosquitoes, we used 122 ssu rDNA sequences representing all known clades of the classical Microsporidia (74), Metchnikovellida (four) and Chytridopsida (one). As close outgroups, we used Rozellomycota (six) and Fungi (28). For details concerning all ingroup and outgroup taxa see Table S2. Sequences were aligned using the l‐ins‐i algorithm in mafft version 7.388 (Katoh et al., 2002; Katoh & Standley, 2013) as implemented in geneious R11.1.5. The final alignment consisted of 2,718 nucleotide positions. The best fit model of DNA evolution (GTR + I + G) was chosen by partitionfinder2 (Lanfear, Calcott, Ho, & Guindon, 2012). Phylogenetic trees were reconstructed using maximum likelihood (ML) in garli version 2.0 (Zwickl, 2006) and Bayesian inference (BI) in mrbayes 3.2.6 (Ronquist et al., 2012). Each BI run of four independent chains was performed in 2 × 10,000,000 generations, and the trees were sampled every 1,000 generations. The final consensus tree was generated after discarding the burn‐in fraction of 0.25% of initial trees; the average standard deviation of split frequencies dropped below .003. Bootstrap support for the ML tree was calculated by using 1,000 data replicates as implemented in garli. The trees were edited in figtree 1.4.4 (Rambaut, 2018) and further in corel draw X4.

### 2.9 Statistical analyses

Sequence reads from microsporidia were normalized via the otutab_rare algorithm (Edgar & Flyvbjerg, 2018) to compare sample diversities. The diversity of OTUs in individual samples were calculated using the alpha_div algorithm (Edgar & Flyvbjerg, 2018). Due to the lack of a near‐normal distribution in any sample (*p* < .05) in the initial Shapiro–Wilk test (Shapiro & Wilk, 1965), the nonparametric Kruskal–Wallis test (Kruskal & Wallis, 1952) was used to compare technical repetition series of spore dilutions and control co‐infections, as well as to compare microsporidian species detected in mosquitoes. Rarefaction curves were generated using past software version 3.23 (Hammer, Harper, & Ryan, 2001). The level of significance of the microsporidian infection frequencies was tested with the Dunn test (Dunn, 1964) constituting the post‐hoc test for Q‐Cochran analysis (Cochran, 1950). A heatmap was prepared using heatmapper tools (Babicki et al., 2016). The chi‐squared test statistic (Pearson, 1900) was used to evaluate whether there is an association between the detected microsporidia and their occurrence in different host species or in mixed infections, and the relationship between numbers of sequence reads per OTU in the metabarcoding approach and a successful amplification of the V1–V3 region. Pearson's correlation coefficient was used to determine the correlation between numbers of reads of individual microsporidians (Pearson, 1895). The results of Pearson's correlation were visualized in gephi software version 0.9.2 (Bastian, Heymann, & Jacomy, 2009).

Microsporidian DNA was considered as incidental in field‐collected mosquitoes when noted in <1% of all analysed individuals, its OTU was covered by fewer than 50 sequences and the species has not been previously reported from mosquitoes.

## 3 RESULTS

### 3.1 Proof‐of‐concept experiment: Positive and negative samples and mini‐COI barcoding of the hosts

Microsporidia‐positive samples including DNA extracts from spores (36), mixture of spores (36) and cultured microsporidia (three), as well as negative controls including blank DNA extractions (three) and blank PCRs (six) yielded 1,413,000 sequence reads after quality filtering. None of the negative control samples passed our sequencing quality threshold of having at least 10 reads per OTU. Sequences generated from the DNAs extracted from cultured microsporidians (~18,500) showed three OTUs that corresponded to the microsporidian species from which DNA samples were extracted: *Anncaliia algerae*, *Vavraia culicis* and *Enterocytozoon bieneusi*.

Almost 106,000 sequence reads were obtained from the series of 10‐fold dilution of *Enterocytozoon cuniculi*, *E. hellem* and *E. intestinalis* spores. Samples containing 100, 1,000 and 10,000 spores/ml were represented by, respectively, 673 (*SD* = 94), 1,499 (*SD* = 407) and 9,524 (*SD* = 862) sequence reads. The method was reproducible for all tested species to the level of 100 spores per 1 ml (*R* ^2^> .99) (Figure S1). The repeatability of the method was supported by the Kruskal–Wallis test (*H* < .006; *p* < .05).

**Figure 2:**
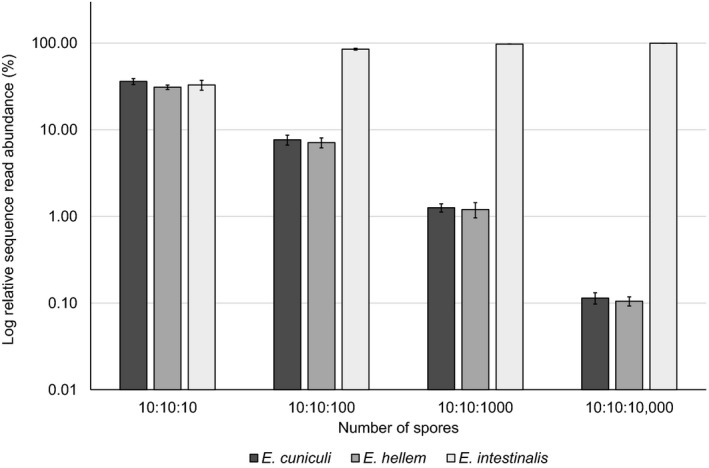
Relationship between the numbers of spores of different species in mixed samples and their relative sequence read abundance in quality‐filtered sequence data. DNAs from *Encephalitozoon cuniculi* and *E. hellem* spores were detected even when they were mixed in a ratio of 1 to 1,000 (10:10:10,000) with *E. intestinalis*

Analysis of the relative sequence read abundance in sequencing results from DNA samples of a mixture of microsporidian species showed that DNAs from species present in smaller quantities in the sample (*E. cuniculi* and *E. hellem* versus *E*. *intestinalis*) were successfully detected in each mixture of spores in all technical replicates (Figure 2). DNAs from *E. cuniculi* and *E. hellem* spores were detected in the sample even when they were mixed in a ratio of 1–1,000 with *E. intestinalis*. In this sample, they were represented by about 0.1% of all reads, which corresponded to the assumed share of the spores representing each species. The repeatability of the experiment was supported by the Kruskal–Wallis test (*H* < .008; *p* < .05).

To test the new method on colony mosquitoes, 213 samples consisting of the V5 region amplicons from *Aedes aegypti*, *Anopheles quadrimaculatus*, *Culex quinquefasciatus* and *Uranotaenia lowii* and negative controls (13) were sequenced in one experiment. Negative controls included PCRs performed on the DNA extracted from preservation medium for mosquitoes, blank DNA extractions and blank PCRs. After quality filtering, 480,307 reads were used in the OTU analysis. None of the negative control samples passed our sequencing quality threshold to generate OTUs. Less than 9% of the whole sequence data were of nonmicrosporidian origin. From these nonmicrosporidian sequences, we reconstructed one OTU showing 96% identity to the acetylcholinesterase (*Ace1*) gene from *Aedes aegypti* (GenBank accession no. BK006052). This OTU was found in almost all mosquito individuals (181/200). However, the number of reads for this OTU never exceeded 785 per individual (median = 312). Among colony mosquitoes, we found microsporidian DNA in two individuals belonging to *Anopheles quadrimaculatus*. The OTU we found in this species matched Microsporidium sp. OB1 (GenBank accession no. MG456597) originally reported from the geometer moth *Operophtera bruceata* (Lepidoptera).

Based on mini‐COI data, all mosquitoes were unambiguously assigned to the correct species. The COI sequence reads in a mosquito individual positive for microsporidia amounted to 7% (*SD* = 4) while the remaining reads represented the V5 region library. This result corresponded to the molar ratio of 1:10 used for pooling the libraries for sequencing.

### 3.2 Applying the metabarcoding method for field‐collected mosquitoes

Using mini‐COI data, all field‐collected mosquitoes were unambiguously assigned to seven species: *Aedes* *cinereus* (13), *Aedes vexans* (19), *Coquillettidia richiardii* (16), *Ochlerotatus annulipes* (63), *O. cantans* (77), *O. punctor* (11) and *O. sticticus* (13) (Table 1; Table S1).

**Table 1:** Microsporidia found in field‐collected mosquitoes using metabarcoding

Microsporidia	Mosquitoes								Prevalence (%) of microsporidian species	
	*Aedes cinereus*	*Aedes vexans*	*Coquillettidia richiardii*	*Ochlerotatus annulipes*	*O. cantans*	*O. punctor*	*O. sticticus*	All		
	92.3%	63.2%	56.3%	54.0%	57.1%	72.7%	61.5%	59.9%	In all	In M‐P
	(*n* = 13)	(*n* = 19)	(*n* = 16)	(*n* = 63)	(*n* = 77)	(*n* = 11)	(*n* = 13)	(*n* = 212)		
*Amblyospora salinaria*							1	1	0.47	0.67
*Amblyospora* sp.				1	1		1	3	1.42	2.00
*Encephalitozoon hellem*					2			2	0.94	1.33
*Enterocytospora artemiae*				1				1	0.47	0.67
Microsporidium sp. PL01	12	10	9	31	27	8	7	104	49.1	69.33
*Nosema adaliae*	1			1		1		3	1.42	2.00
*N. ceranae*					2			2	0.94	1.33
*N. chrysorrhoeae*/*portugal*		2	1	4	16			23	10.8	15.33
*N. pieriae*			1	2				3	1.42	2.00
*N. thomsoni*		1						1	0.47	0.67
*Nosema* sp. CHW−2007a		2	1	2	1		1	7	3.30	4.67

In total, the V5 region library of the field‐collected mosquitos (212) and negative controls (five) generated about 1,330,000 reads after quality filtering. Negative controls yielded no sequence data using our default threshold. In microsporidia‐free mosquitoes the number of reads never exceeded 626 per sample (median = 101). Together, nonmicrosporidian sequences accounted for only 12% of all quality filtered reads; most of them were of host origin (11.9%) and coded ssu rRNA genes (9.8%) or a collagen alpha‐1 chain gene (2.1%; 100% identity with GenBank accession no. XM_021840458). The less abundant OTUs represented ssu rDNA fragments from fungi (in three mosquito individuals; <.03% of sequence reads), gregarine (in one individual; <0.002%) and human (in one individual; <0.001%) (Table S1).

In field‐collected mosquitoes, microsporidian DNA was found in almost 60% (127/212) of individuals representing all analysed species (Table 1). The OTU clustering across microsporidia‐positive samples produced 11 unique OTUs that represented: a new microsporidian ssu rDNA gene sequence which we named Microsporidium sp. PL01, *Amblyospora salinaria*, *Amblyospora* sp. (identical to AY090055), *Encephalitozoon hellem*, *Enterocytospora artemiae*, *Nosema adaliae*, *N. ceranae*, *N. pieriae*, *N. thomsoni*, *Nosema* sp. CHW‐2007a, and indistinguishable in this sequence fragment *N. chrysorrhoeae* and/or *N*. *portugal* (Table 1; Table S1). Rarefaction curve analysis showed that the read depth was sufficient to recover all microsporidian species in the tested individuals (Figure S2). The analysis revealed that 10,000 reads per sample is required to identify all microsporidian diversity in the tested host.

Except for *A. cinereus* and *O. punctor* that hosted two microsporidian species, all the remaining mosquito species were positive for at least four different microsporidians that could potentially infect them (Table 1). The highest richness of different Microsporidia was observed in *O. annulipes*; mosquitoes belonging to this species potentially hosted six different microsporidians: Microsporidium sp. PL01, *Amblyospora salinaria* and at least four species from the genus *Nosema*. The presence of *E. hellem*, *E. artemiae* and *N. ceranae* was recognized as incidental because each of the OTUs was noted only in one host species and was found in <1% of all mosquito individuals.

**Figure 3:**
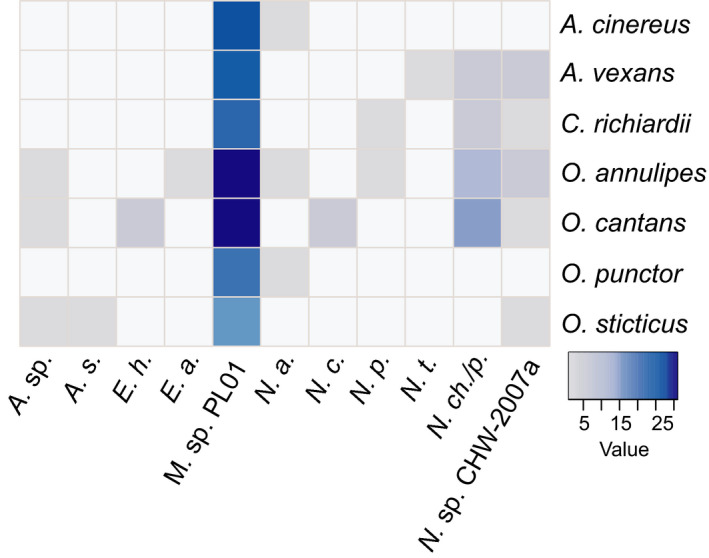
Heatmap showing microsporidian species detected in field‐collected mosquitoes. Blue colour darkens as the number of infected individuals in each mosquito species increases. Abbreviations of microsporidian species: *A*. sp., *Amblyospora* sp.; *A*. *s*., *Amblyospora salinaria*; *E*. *h*., *Encephalitozoon hellem*; *E*. *a*., *Enterocytospora artemiae*; M. sp. PL01, Microsporidium sp. PL01; *N*. *a*., *Nosema adaliae*; *N*. *c*., *N. ceranae*; *N*. *p*., *N. pieriae*; *N*. *t*., *N. thomsoni*; *N*. *ch*./*p*., *N. chrysorrhoeae* and/or *N*. *portugal*; *N*. sp., *Nosema* sp. CHW‐2007a [Colour figure can be viewed at wileyonlinelibrary.com]

The predominant microsporidium that was noticed in all mosquito species was Microsporidium sp. PL01 (69.3% of all infected individuals) (Table 1). This species occurred significantly more often than any of the other microsporidians (*p* < .01) in each of the analysed mosquito species (Figure 3); Berger–Parker (.69) and Simpson (.51) dominance indexes supported this observation. In addition, a high frequency of *N. chrysorrhoeae* and/or *N*. *portugal* was observed (15.3%, *p* < .05), especially in *O. cantans*; this microsporidian DNA was also detected in mosquitoes belonging to *Aedes vexans*, *C. richiardii* and *O. annulipes*. The third most common detected microsporidian (4.7%) was *Nosema* sp. CHW‐2007a, which was recorded in five of seven examined host species (Figure 3). In the remaining possible microsporidian pairs, there were no significant differences in their frequency (*p *> .05).

**Figure 4:**
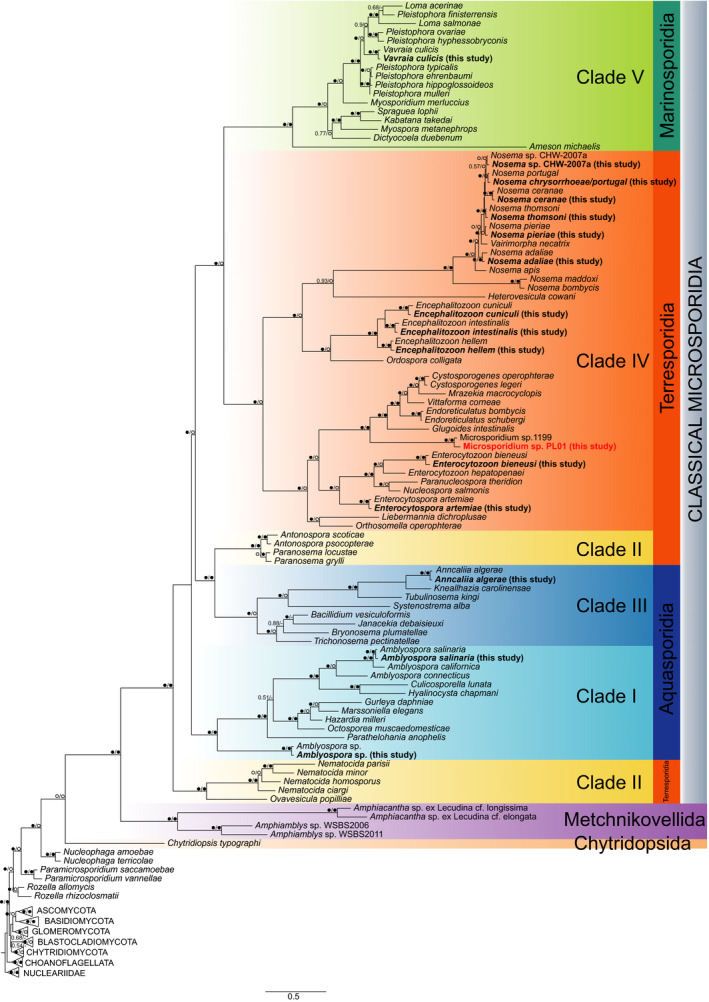
Phylogenetic tree of Microsporidia inferred from BI and ML analyses of concatenated ssu rDNA sequence data. Values near branches show Bayesian posterior probabilities (PP) and bootstrap support values (BS) (PP/BS). Black circles: maximally supported; empty circles: supported > .95 PP and > 75% BS. Sequences found in this study are in bold; the new species found in this study is in red [Colour figure can be viewed at wileyonlinelibrary.com]

Results of the phylogenetic analysis showed that Microsporidium sp. PL01 forms clade with Microsporidium sp. 1199 (GenBank accession no. FN610845.1) and represents another species in the same genus nested in the microsporidian Clade IV (Figure 4).

### 3.3 Co‐occurrence of different Microsporidia in single mosquito individuals

The co‐occurrence of DNAs representing different microsporidian species in one host individual was recorded in 20 field‐collected mosquitoes (9.4%); this relationship concerned all microsporidian and host species (Table 2). There were no statistically significant relationships between co‐occurring microsporidians and mosquito species (χ^2^ = 1.2; *p *> .05). Microsporidia considered as incidental—*E. hellem*, *E. artemiae* and *N. ceranae*—were found with the most abundant Microsporidium sp. PL01 in four mosquito individuals; additionally, in a single case, *N. ceranae* was noted with *N. chrysorrhoeae* and/or *N*. *portugal*. The co‐occurrence rate remained high in all host species, even omitting the incidental microsporidians. More than two microsporidian species were found in 12.6% of all microsporidia‐positive mosquitoes.

**Table 2:** Numbers of sequence reads representing microsporidian OTUs in co‐infected mosquitoes

Sample ID	Mosquito species	*A*. *s*.	*A*. sp.	*E*. *h*.	*E*. *a*.	M. sp. PL01	*N*. *a*.	*N*. *c*.	*N*. *ch*./*p*.	*N*. *p*.	*N*. *t*.	*N*. sp.
AT.p01.C08	*O. annulipes*	–	–	–	–	–	–	–	100	118	–	–
AT.p01.D08	*O. annulipes*	–	–	–	–	–	–	–	16	20	–	–
AT.p01.H10	*Aedes vexans*	–	–	–	–	38	–	–	584	–	–	–
AT.p02.A12	*O. annulipes*	–	–	–	–	15	–	–	15	–	–	–
AT.p02.C06	*O. cantans*	–	–	–	–	544	–	16	–	–	–	–
AT.p02.C07	*O. cantans*	–	–	31	–	6,766	–	–	–	–	–	–
AT.p02.D06	*O. cantans*	–	–	–	–	–	–	–	25	–	–	21
AT.p02.D12	*O. annulipes*	–	–	–	30	19	–	–	–	–	–	–
AT.p02.E06	*O. annulipes*	–	–	–	–	**1,283**	–	–	**3,080**	–	–	**73**
AT.p02.F05	*O. cantans*	–	14	–	–	426	–	–	–	–	–	–
AT.p02.F09	*O. cantans*	–	–	–	–	–	–	19	21	–	–	–
AT.p02.H09	*C. richiardii*	–	–	–	–	22	–	–	19	–	–	22
AT.p03.B06	*O. annulipes*	–	–	–	–	1647	118	–	–	–	–	15
AT.p03.C03	*O. punctor*	–	–	–	–	18	56	–	–	–	–	–
AT.p03.D05	*O. sticticus*	–	–	–	–	22	–	–	–	–	–	16
AT.p03.D07	*Aedes vexans*	–	–	–	–	**8,000**	–	–	–	–	**2,948**	**19**
AT.p03.D08	*A. cinereus*	–	–	–	–	3,924	141	–	–	–	–	–
AT.p03.E05	*O. cantans*	–	–	17	–	503	–	–	–	–	–	–
AT.p03.E07	*O. sticticus*	24	21	–	–	–	–	–	–	–	–	–
AT.p03.F04	*C. richiardii*	–	–	–	–	372	–	–	–	59	–	–

Samples that were unreadable in direct Sanger sequencing of V1‐V3 amplicons are in bold; samples where direct Sanger sequencing of V1‐V3 amplicons detected only Microsporidium sp. PL01 are marked with a grey background.

Abbreviations of microsporidian species: *A*. *s*. — *Amblyospora salinaria*, *A*. sp. — *Amblyospora* sp., *E*. *h*. — *Encephalitozoon hellem*, *E*. *a*. — *Enterocytospora artemiae*, M. sp. PL01 — Microsporidium sp. PL01, *N*. *a*. — *Nosema adaliae*, *N*. *c*. — *N. ceranae*, *N*. *ch*./*p*. — *N. chrysorrhoeae* and/or *N*. *portugal*, *N*. *p*. — *N. pieriae*, *N*. *t*. — *N. thomsoni*, *N*. sp. — *Nosema* sp. CHW‐2007a.

Although most microsporidian co‐infections occurred with the most abundant Microsporidium sp. PL01 (76.2%), the highest convergence rate of co‐infections concerned microsporidians of the genus *Nosema*. Among 39 cases of the presence of *Nosema* DNAs in mosquitoes, 23 of them (59%) were observed in co‐occurrence with DNA representing the other microsporidian species. The chi‐squared statistic showed that *Nosema* spp. occurred more frequently in mosquitoes with another microsporidian species than individually (χ^2^ = 25.15; *p* < .05).

**Figure 5:**
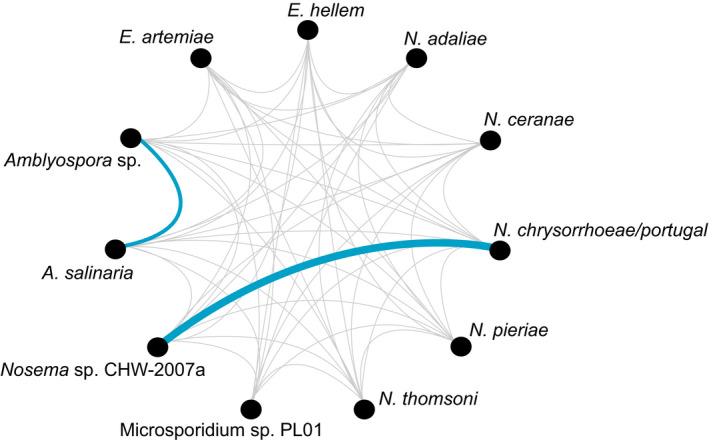
Correlation network between the numbers of reads representing each microsporidian species found in co‐infected mosquitoes. Blue lines—statistically significant correlation. Almost perfect correlation (thick blue line) was observed when *Nosema chrysorrhoeae*/*portugal* occurred in mixed infection with *Nosema* sp. CHW‐2007a. High correlation (thin blue line) was found between co‐infecting *Amblyospora* sp. and *Amblyospora salinaria* (for correlation values see Table S3) [Colour figure can be viewed at wileyonlinelibrary.com]

A statistically significant relationship (*p* < .05) was found between the numbers of reads representing a given co‐infecting microsporidian species. An almost perfect correlation was observed in increasing numbers of sequence reads between *N. chrysorrhoeae*/*portugal* and *Nosema* sp. CHW‐2007a (*r* = .9); also, a similarly high correlation was observed between *Amblyospora* sp. and *Amblyospora salinaria* (*r* = .51) (Figure 5; Table S3).

### 3.4 Comparison of metabarcoding and standard molecular approach

**Figure 6:**
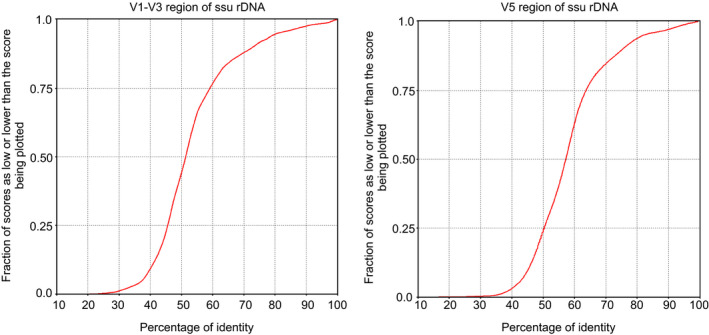
Results of Kolmogorov–Smirnov tests for the microsporidian V1–V3 and V5 sequence alignments. The plots show that about .75 of the V1–V3 and V5 aligned sequences had less than 60% and 64% identical nucleotide positions, respectively, which means that the variation in the nucleotide sequence of both markers is similar [Colour figure can be viewed at wileyonlinelibrary.com]

The Kolmogorov–Smirnov plots for the microsporidian V1–V3 and V5 sequence alignments showed that the proposed V5 marker is only 4% less variable than the standard sequence covering V1–V3 regions (about 40% and 36% of different nucleotide positions, respectively) (Figure 6). The extraction of duplicate reads from the alignment comprising the V5 region representing 607 microsporidian species belonging to 120 genera indicated that the marker enables the proper identification of almost all species in the data set (596/607, >98%). Several sequences that were found in duplicates represented species mainly of the genus *Nosema* (e.g., *N*. *chrysorrhoeae* and *N*. *portugal* or a group consisting of *N. antheraeae, N. trichoplusiae, N. spodopterae*, and *N. philosamiae* showed no variation in the V5 region). However, in these sample groups extremely low or no variation was also observed in other regions of the ssu rDNA, including V1–V3 marker (data not shown). In three cases, the same V5 region sequences were found in species representing different genera (*Conglomerata obtusa* and *Berwaldia schaefernai*; *Ameson portunus*, *Ameson pulvis* and *Nadelospora canceri*; *Tetramicra brevifilum* and *Microgemma caulleryi*).

In total, 42 microsporidia‐positive samples were obtained using the standard V1F/530R primer set and DNA extracts from field‐collected mosquitoes previously used to test the metabarcoding method (Figure S3, Table S1). Using the same DNA templates we successfully amplified a full‐length COI‐barcode (about 670 bp), which means that all DNA isolates were suitable for PCR amplification (Figure S4). As PCR products were found among 127 microsporidia‐positive mosquitoes identified previously by the metabarcoding method, the standard PCR approach gave almost 67% false‐negative samples (Table S4). We found a statistically significant correlation (χ^2^ = 68.59, *p* < .05) between the detection of microsporidia using the standard molecular approach and obtaining at least 300 reads per OTU found by the metabarcoding method. Direct Sanger sequencing of the V1–V3 amplicons revealed two samples with unreadable chromatograms, one sample positive for *Amblyospora* sp. which was in agreement with the metabarcoding result, and 39 samples positive for Microsporidium sp. PL01. The Microsporidium sp. PL01 populations infecting different mosquito host species shared the same V1–V3 sequence. Two unreadable chromatograms were found in mosquitoes that were highly co‐infected with Microsporidium sp. PL01 and two *Nosema* spp. (samples AT.p02.E06 and AT.p03.D07, bold type in Table 2). On the other hand, direct V1–V3 amplicon sequencing failed to show four mixed samples where Microsporidium sp. PL01 dominated significantly over other co‐infecting species (highlighted in grey in Table 2).

## 4 DISCUSSION

### 4.1 Effectiveness of metabarcoding microsporidia and their hosts

The metabarcoding approach proposed in this study can be used for fast, accurate and sensitive detection of microsporidia in all types of DNA samples. Our data show that the new marker covering the hypervariable V5 region of ssu rDNA allows correct identification of almost all classical microsporidian species known from rDNA sequence data.

The V5 region is flanked by group‐conserved sequences that allowed us to design the CM‐V5F/CM‐V5R primer set with great specificity towards microsporidian ssu rDNA. Indeed, the specificity of our primers seems to be higher than the commonly used V1F/530R pair applied by Williams et al. (2018), because in our experiments, the nontarget sequences never exceeded 12% of quality‐filtered sequence data. Results of our phylogenetic analysis, which included the V5 region sequences found in this study, support that the new primer set enables the amplification of ssu rDNA in different microsporidian evolutionary lineages, including Aquasporidia, Terresporidia and Marinosporidia (Vossbrinck & Debrunner‐Vossbrinck, 2005) (Figure 4). However, recently published first sequence data for the two remaining microsporidian lineages—the metchnikovellid *Amphiamblys* sp. (Galindo et al., 2018; Mikhailov, Simdyanov, & Aleoshin, 2017) and chytridiopsid *Chytridiopsis typographi* (Corsaro et al., 2019)—suggest that the CM‐V5F/CM‐V5R primer set is rather group‐specific for the classical Microsporidia. This indicates that there may be a need to develop additional primers that will be group‐specific for both the Metchnikovellida and the Chytridiopsida to study species diversity in the whole phylum.

Our metabarcoding approach allowed us to detect 100 spores/ml, which is comparable with other PCR‐based methods (Menotti et al., 2003; Rinder et al., 1998; Wolk et al., 2002) or microarray techniques developed for *Encephalitozoon* spp. in clinical samples (Wang et al., 2005). Usually, clinical samples are not accessible; therefore, the screening of different microsporidian species in the same sample at the same time may be of clinical benefit. The advantage of the metabarcoding is that it allows simultaneous screening of all species, without the need to detect particular species in separate PCRs. For example, methods commonly used for microsporidia detection in water are based on spore staining in smears from concentrated water samples, followed by the PCR amplification of marker sequences using species‐specific primers (e.g., Ben Ayed et al., 2012; Izquierdo et al., 2011; Li et al., 2012). The metabarcoding approach could help overcome these difficulties, reducing the time and costs of the analysis. In addition, the metabarcoding of concentrated water samples could be standardized, which can help to develop good laboratory methods for more accurate waterborne disease risk assessment.

The comparison of our new method with a standard PCR‐based approach clearly shows that metabarcoding is more sensitive and accurate in detecting microsporidian infections. Our results show that direct amplicon sequencing would be impractical in co‐infected samples where one microsporidian species was present at a much higher level of infection than the other co‐infecting species. Although amplicon cloning in a plasmid vector and subsequent Sanger sequencing of several clones can detect mixed samples in the cases of relatively balanced co‐infections, the dominance of one microsporidian species would require sequencing a large number of clones, which is not usually performed.

Based on the same mosquito DNA samples, we successfully amplified both the full‐length COI‐barcode (about 670 bp) and the mini‐COI fragment (373 bp) for metabarcoding. *COI* sequence analysis showed that both fragments allowed us to unambiguously assign all mosquitoes to the correct species. Therefore, the mini‐COI fragment can be successfully used in NGS approaches to determine microsporidian host species.

### 4.2 Microsporidia in field‐collected mosquitoes

More than half of the field‐collected mosquitoes analysed in this study were positive for microsporidian DNA. Reports of the prevalence of microsporidia in adult mosquito populations in nature are sparse and focused on particular microsporidians and their primary mosquito hosts. In one experiment conducted for almost 20 years, the prevalence of *Amblyospora stimuli* infections in *Aedes stimulans* adult female populations was relatively low and ranged from 1% to 9.6% (Andreadis, 1999). On the other hand, in the study concerning the life cycle and ecology of *Amblyospora khaliulini*, prevalence rates of *Amblyospora khaliulini* infections in adult mosquito females ranged from 16.4% to 50% (Andreadis, Thomas, & Shepard, 2018). In our study, all mosquito species showed a much higher rate of microsporidian‐positive individuals, ranging from 54.0% to 92.3% (Table 1). However, in the studies conducted by Andreadis et al. (1999, 2018), Microsporidia were identified by microscopic examination of infected tissues; mosquitoes were considered to be infected if either the vegetative or spore developmental stage of the parasite was observed.

The studies of Andreadis (1999), Andreadis et al. (2018) were based on a single collection of mosquitoes during the end of April or May, which are not the warmest months of the year in the studied areas (public data of the National Weather Service, National Oceanic and Atmospheric Administration; www.weather.gov). For the present study, mosquitoes were collected in July and August, which are the warmest months in Poland (public data of the National Research Institute, Institute of Meteorology and Water Management; www.imgw.pl). Our preliminary results of a phenological survey of microsporidian infections in mosquito natural populations suggest that during the warmest months of the year the prevalence of microsporidians is higher than in other seasons (about 30%) (Trzebny A., Liberska J., Dabert M. in prep.) and can reach about 60% of microsporidia‐positive individuals. Thus, the differences between prevalence noted in our and the previous studies could result from both the season of mosquito capture and the use of a much more sensitive method for microsporidia detection.

The presence of microsporidian DNA does not necessarily result from infection. Therefore, we excluded *E. hellem*, *E. artemiae* and *N. ceranae* as infecting factors because they had been noted in fewer than 1% of all analysed individuals and their OTUs were covered by low numbers of reads. However, there is no empirical basis to exclude *Nosema* spp. or *Amblyospora* spp. even though in the present study we recorded them in single host individuals and/or their OTUs were represented by low numbers of reads. To our knowledge, the remaining microsporidian species, found by us in field‐collected mosquitoes, have been recorded in Culicidae for the first time.

Our observations based on numbers of reads representing the newly recorded microsporidian species OTUs or their occurrence in multiple mosquito individuals support the hypothesis that these species can indeed infect mosquitoes. A wide range of hosts for members of the genus *Nosema* also supports this hypothesis. For example, *N. thomsoni* has been noted in *Andrena vaga* (Hymenoptera) (Ravoet et al., 2014), *Harmonia axyridis* (Coleoptera) (Vilcinskas, Stoecker, Schmidtberg, Röhrich, & Vogel, 2013) and *Choristoneura conflictana* (Lepidoptera) (Kyei‐Poku, Gauthier, & Van Frankenhuyzen, 2008).

Among the other microsporidians detected in our study, *N. adaliae* was found in *Adalia bipunctata* (Coleoptera) (Steele & Bjørnson, 2014), while *N. pieriae*, *N. chrysorrhoeae*, *N. portugal* and *Nosema* sp. CHW‐2007a were noted in different lepidopteran hosts (Huang et al., 2008; Hyliš et al., 2006; Maddox et al., 1999; Yaman, Bekircan, Radek, & Linde, 2014). *Amblyospora* sp. recorded in our study had been previously found in *Cyclops strenuous* (Copepoda) from the Czech Republic (Vossbrinck et al., 2004). The dominant presence of one microsporidian species—Microsporidium sp. PL01—in all mosquito species analysed in this study suggests that there may be no "primary host" in microsporidia–mosquito relationships and the observed prevalence reflects only the abundance of a generalist microsporidian species infecting mosquito larvae in the water environment. Nevertheless, ultrastructural assessment, based on histology and transmission electron microscopy, should be carried out to confirm the actual infection.

The new microsporidian ssu rDNA sequence found in this study is most similar to that of Microsporidium sp. 1199, which was recorded in freshwater populations of *Gammarus duebeni* (Crustacea, Amphipoda) in a rivulet from Holyhead Island, Wales, UK, during research into the molecular characterization of the microsporidians of this host across its natural range (Krebes et al., 2010). Our phylogenetic analysis results strongly support that Microsporidium sp. PL01 and Microsporidium sp. 1199 represent different species in the same genus, which belongs to one of the two main clades in the Terresporidia. Members of this clade mostly parasitize terrestrial arthropods, but this clade also groups microsporidians that can infect vertebrates, including humans (e.g., *Enterocytozoon bieneusi*).

### 4.3 Microsporidia in co‐infections

Our metabarcoding method allowed us to detect a relatively high level (9.4%) of co‐occurrence of DNAs representing different microsporidian species in the same host individual. We cannot exclude accidental intake of spores in food; however, high numbers of sequence reads representing *N*. *thomsoni* or *N*. *chrysorrhoeae*/*portugal* in samples where they co‐occurred with Microsporidium sp. PL01 are evidence of mixed infection by these species.

It is noteworthy that we found *Nosema* spp. in field‐collected mosquitoes mostly in co‐infections. Furthermore, we noted that three out of five *Nosema* species—*N. adaliae*, *N. pieriae* and *N. thomsoni*—in our study were found only in co‐infections. Additionally, we found a positive correlation between numbers of reads for OTUs representing *N*. *chrysorrhoeae* and/or *N*. *portugal* and *Nosema* sp. CHW‐2007a, which suggests that these species support each other. A similar relationship was found for the *Amblyospora* spp. where the occurrence of *Amblyospora salinaria* was associated with the occurrence of *Amblyospora* sp., although in this case the numbers of reads suggest a lower level of infection. However, quantitative analyses should be carried out to confirm these relationships. To conclude, we believe that our observations based on the microsporidian DNA occurrences reflect the actual microsporidian diversity in a tested sample; however, the hypotheses concerning infections and mutual relationships of co‐infecting species should be tested using a more intensive host sampling with the use of ultrastructural data.

## 5 CONCLUSIONS

We have proposed a new molecular approach for the detection of microsporidian DNA in samples extracted from their hosts. The method uses NGS sequence data from the hypervariable V5 region of the ssu rDNA and the shortened fragment of the standard metazoan DNA‐barcode (i.e., the mitochondrial *COI* gene) for microsporidian and host species identification, respectively.

We have tested this metabarcoding approach on microsporidians infecting mosquitoes; however, the method can be applied to all types of DNA extracts, including clinical and environmental samples. We compared our new method with the standard molecular approach for microsporidian DNA detection, which uses end‐point PCR and Sanger sequencing. This comparison showed that the metabarcoding is more sensitive and accurate than the traditional method.

An additional advantage of the new method is its effectiveness in identifying microsporidian species that co‐infect the same host individual. Moreover, our data concerning numbers of sequence reads for the microsporidian OTUs found in co‐infected hosts suggest that the occurrence of some microsporidian species in mixed infections could benefit them; however, this observation should be retested using a more intensive host sampling.

Our results show that next‐generation biodiversity assessment is a rapid and cost‐effective method for deciphering sample diversity in greater resolution, including the hidden biodiversity that may be overlooked in approaches based on classical methodology. In addition, our data indicate that information about the relative abundance of a specific pathogen species (number of reads for a specific OTU) obtained by use of the NGS approach can help to discover new ecological relationships among parasite species co‐infecting the same host.

## AUTHOR CONTRIBUTIONS

A.T., A.S‐K. and M.D. designed the research; A.S‐K., J.J.B. and N.S. provided the material; A.T. performed experiments and analysed the data; A.T. and M.D. co‐wrote the first draft of the manuscript. All authors interpreted results and contributed to the final manuscript.

## Supporting information

Supplementary Material

## Data Availability

Sequences generated in this study are available in GenBank under accession nos. MT001301–MT001427, MT015707–MT015901 and MT075548–MT075550. Additional details are available in Figures S1–S4 and Tables S1–S4. Sequence alignments and the remaining data that support the findings of this study are available from the corresponding author upon request.
