# Supplementary material for: A new method of metabarcoding Microsporidia and their hosts reveals high levels of microsporidian infections in mosquitoes (Culicidae)
Source: Mol Ecol Resour. 2020 Jul 1;20(6):1486–504. doi: 10.1111/1755-0998.13205 (PMC7818484; doi:10.1111/1755-0998.13205)
Supplement: Supplementary file 1 — Supplementary Material [file MEN-20-1486-s001.pdf]

MOLECULAR ECOLOGY RESOURCES

Supplemental Information for:

A new method of metabarcoding Microsporidia and their hosts reveals high levels of microsporidian infections in mosquitoes (Culicidae)

Artur Trzebny[1*], Anna Slodkowicz-Kowalska[2], James J. Becnel[3], Neil Sanscrainte[3], Miroslawa Dabert[1]

[1] Molecular Biology Techniques Laboratory, Faculty of Biology, Adam Mickiewicz University, Poznan, Poland

[2] Department of Biology and Medical Parasitology, Faculty of Medicine I, University of Medical Sciences, Poznan, Poland

[3] USDA Agricultural Research Service, Center for Medical Agricultural and Veterinary Entomology, Gainesville, Florida, USA

*** Corresponding author:**

Artur Trzebny: Molecular Biology Techniques Laboratory, Faculty of Biology, Adam Mickiewicz University, Poznan, Poland, E-mail: arturtrzebny@amu.edu.pl

Table of Contents:

Supporting Information	Page
Figure S1	Page 3
Figure S2	Page 4
Figure S3	Page 5
Figure S4	Page 6
Table S1	Page 7
Table S2	Page 12
Table S3	Page 15
Table S4	Page 16

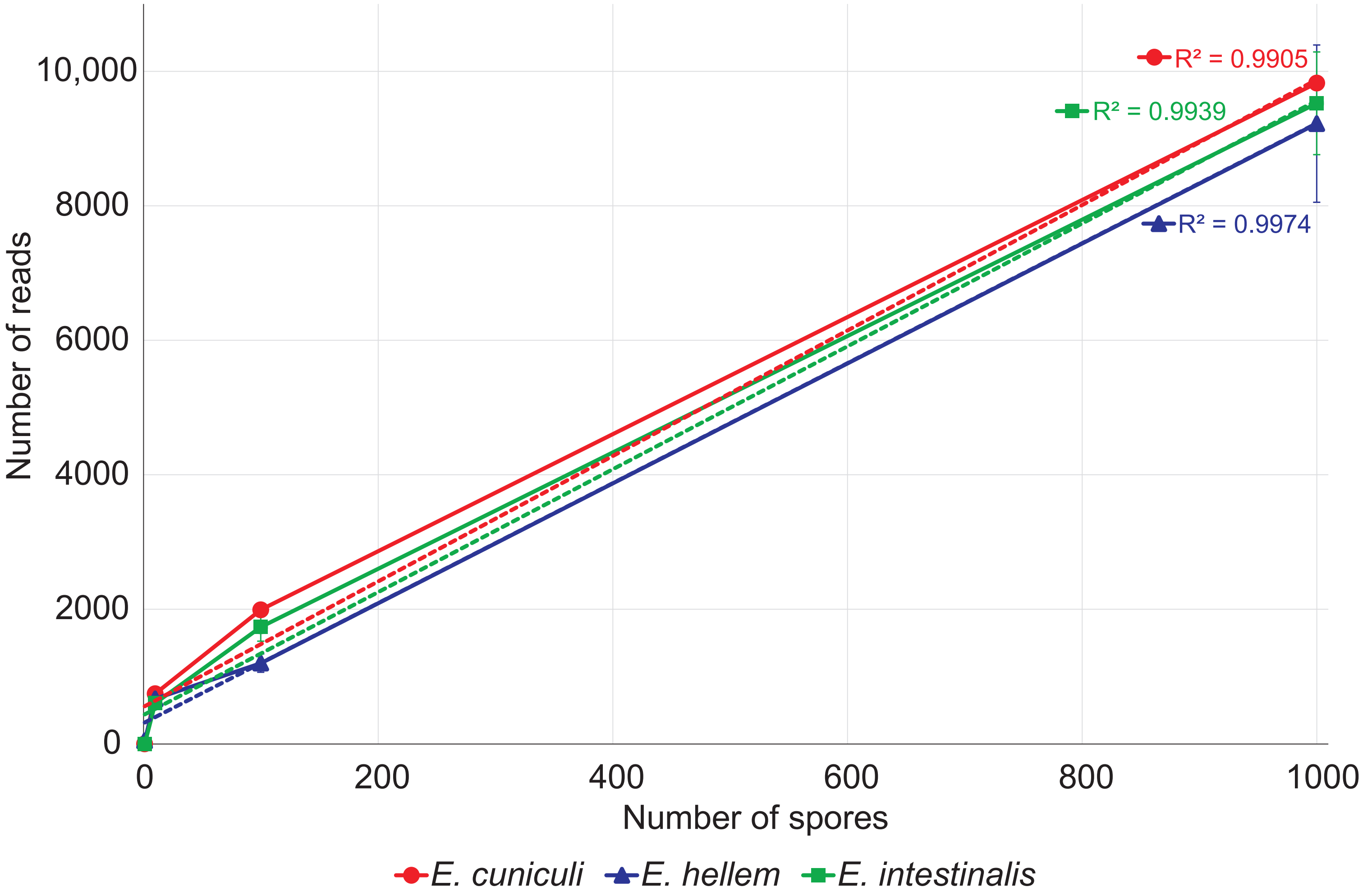

Figure S1. Relationship between the numbers of microsporidian spores used for DNA extraction and the numbers of reads in quality-filtered sequence data. The method was reproducible for all tested species to the level of 100 spores per 1 mL.

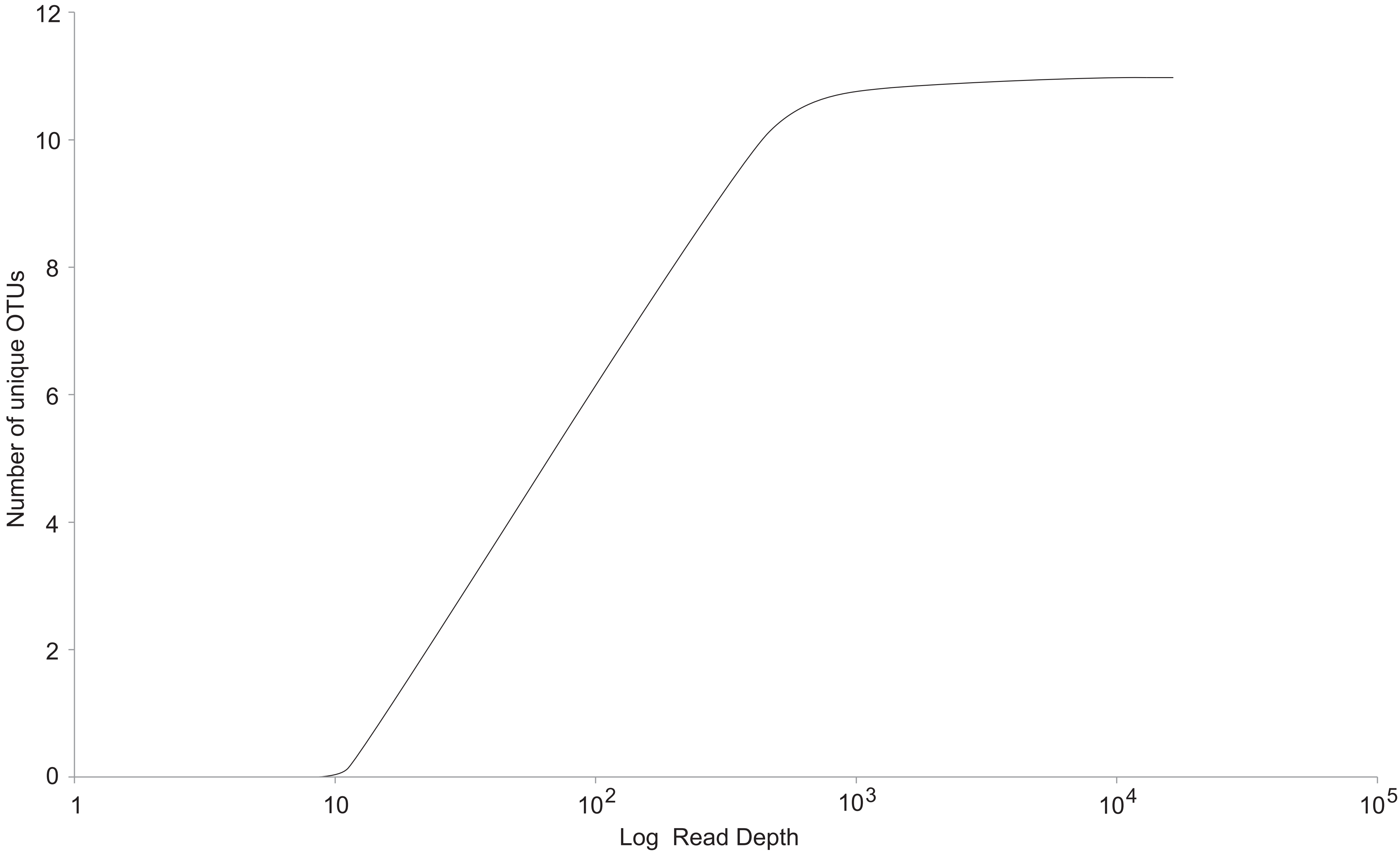

Figure S2. Rarefaction curve showing the number of microsporidian OTUs according the number of sequence reads sampled in field-collected mosquitoes.

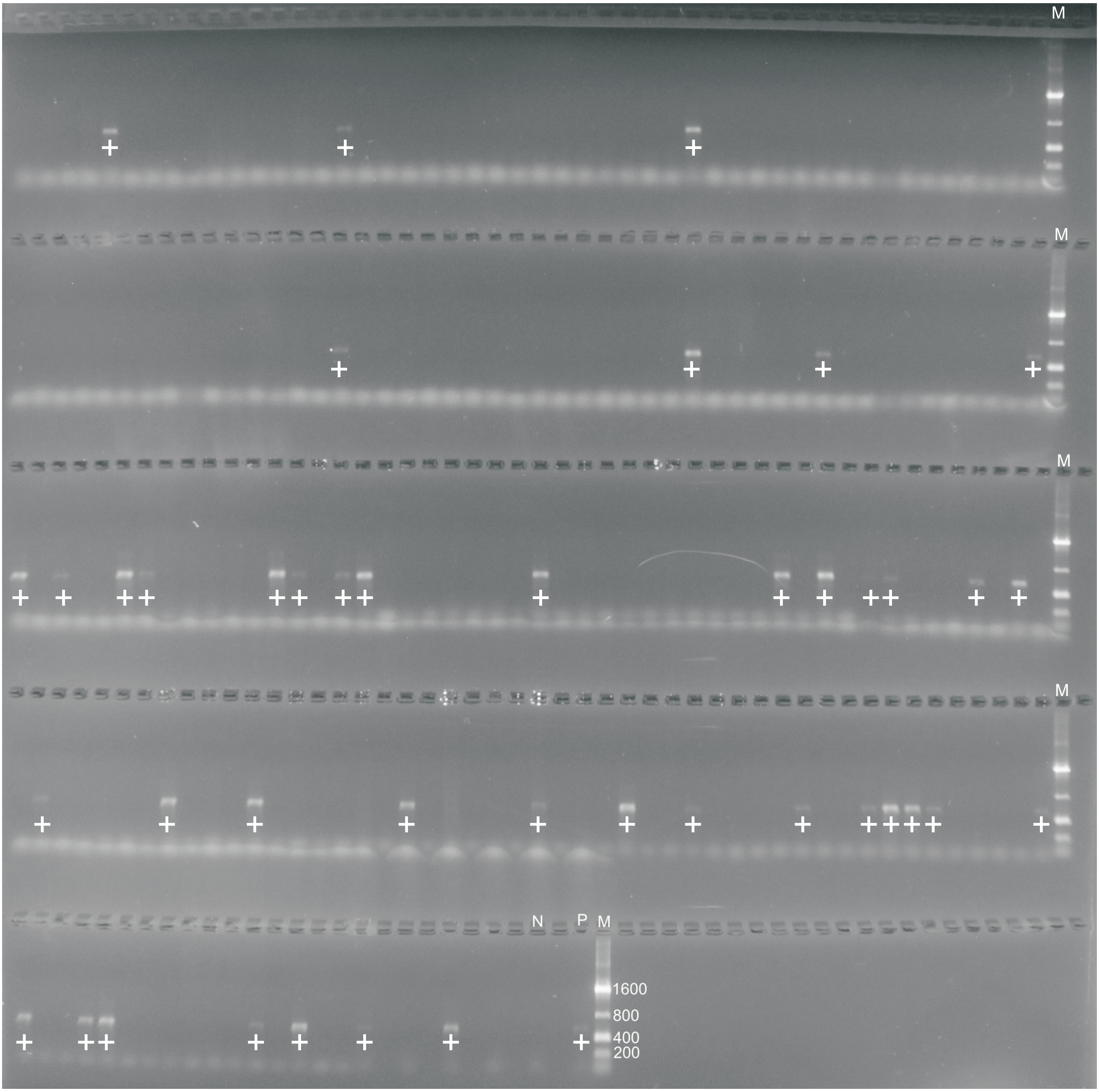

Figure S3. An agarose gel stained with GelRed visualizing of ~400-bp fragments of the V1 – V3 region of ssu rDNA amplified with V1F and 530R primers using DNAs extracted from field-collected mosquitoes. Samples were loaded on the gel as listed in Table S4, alternately every 8 lanes. M. – DNA weight marker, N – negative control, P – positive control (*E. intestinalis* lysate obtained from 100 spores per mL).

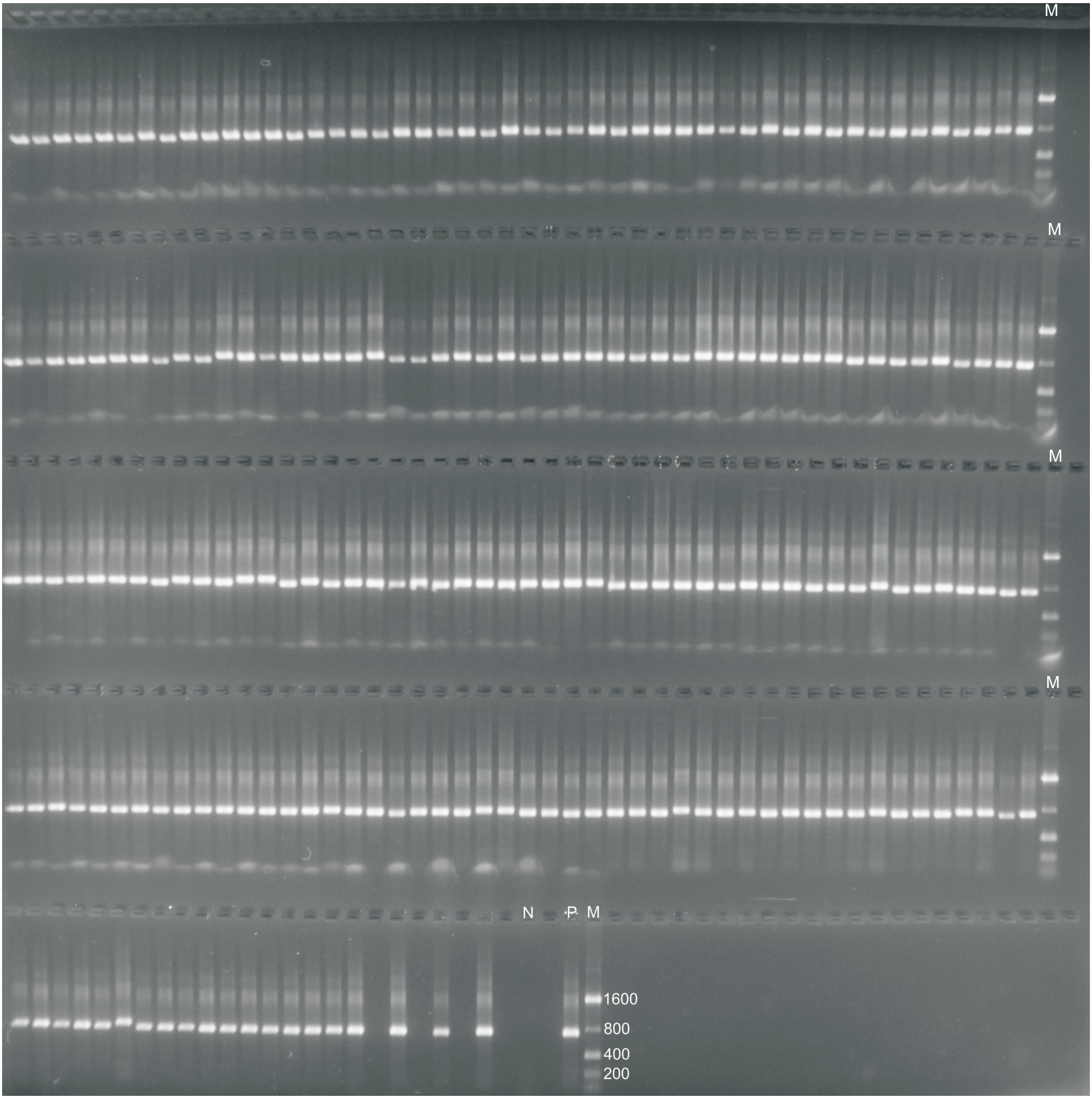

Figure S4. An agarose gel stained with GelRed visualizing of ~670-bp fragments of the full-length COI-barcode amplified with bcdF01 and bcdR04 primers using DNAs extracted from field-collected mosquitoes. Samples were loaded on the gel as listed in Table S4, alternately every 8 lanes. M. – DNA weight marker, N – negative control, P – positive control (*A. aegypti* lysate).

Table S1. GenBank accession numbers of the COI, V1-V3 and V5 ssu rDNA sequences found in this study.

Sample ID	Mosquito species	GenBank no.	OTU	GenBank no.	
		COI		V1 - V3 ssu rDNA	V5 ssu rDNA
AT.p01.G05	*O. sticticus*	MT001301	Microsporidium sp. PL01	n/a	MT015754
AT.p01.A06	*O. sticticus*	MT001302	Microsporidium sp. PL01	MT015707	MT015755
AT.p01.B06	*A. vexans*	MT001303	*Nosema* sp. CHW-2007a	n/a	MT015890
AT.p01.F06	*A. vexans*	MT075548	Uncultured fungus	n/a	MT015898
AT.p01.H06	*O. cantans*	MT001304	Microsporidium sp. PL01	n/a	MT015756
AT.p01.F07	*O. sticticus*	MT001305	Microsporidium sp. PL01	MT015708	MT015757
AT.p01.G07	*O. annulipes*	MT001306	Microsporidium sp. PL01	n/a	MT015758
AT.p01.A08	*O. annulipes*	MT001307	Microsporidium sp. PL01	n/a	MT015759
AT.p01.B08	*O. cantans*	MT001308	*Nosema chrysorrhoeae/portugal*	n/a	MT015867
AT.p01.C08	*O. annulipes*	MT001309	*N. pieriae*; *Nosema chrysorrhoeae/portugal*	n/a	MT015864; MT015868
AT.p01.D08	*O. annulipes*	MT001310	*N. pieriae*; *Nosema chrysorrhoeae/portugal*	n/a	MT015865; MT015869
AT.p01.E08	*O. cantans*	MT001311	Microsporidium sp. PL01	n/a	MT015760
AT.p01.H08	*O. cantans*	MT001312	*Nosema chrysorrhoeae/portugal*	n/a	MT015870
AT.p01.F09	*O. cantans*	MT001313	Microsporidium sp. PL01	MT015709	MT015761
AT.p01.G10	*O. cantans*	MT001314	*Nosema chrysorrhoeae/portugal*	n/a	MT015871
AT.p01.H10	*A. vexans*	MT001315	Microsporidium sp. PL01; *Nosema chrysorrhoeae/portugal*	n/a	MT015762; MT015872
AT.p01.G11	*O. cantans*	MT001316	*Nosema chrysorrhoeae/portugal*	n/a	MT015873
AT.p01.G12	*O. cantans*	MT001317	*Nosema chrysorrhoeae/portugal*	n/a	MT015874
AT.p02.F01	*O. annulipes*	MT001318	*Amblyospora* sp.	MT015710	MT015748
AT.p02.G01	*O. cantans*	MT001319	*Nosema chrysorrhoeae/portugal*	n/a	MT015875
AT.p02.C02	*O. cantans*	MT001320	*Nosema chrysorrhoeae/portugal*	n/a	MT015876
AT.p02.B03	*O. annulipes*	MT001321	Microsporidium sp. PL01	n/a	MT015763
AT.p02.C03	*O. cantans*	MT001322	*Nosema chrysorrhoeae/portugal*	n/a	MT015877
AT.p02.E03	*C. richiardii*	MT001323	Microsporidium sp. PL01	n/a	MT015764
AT.p02.F03	*O. annulipes*	MT001324	Microsporidium sp. PL01	MT015711	MT015765

Sample ID	Mosquito species	GenBank no.	OTU	GenBank no.	
		COI		V1 - V3 ssu rDNA	V5 ssu rDNA
AT.p02.H03	*A. cinereus*	MT001325	Microsporidium sp. PL01	n/a	MT015766
AT.p02.A04	*O. cantans*	MT001326	Microsporidium sp. PL01	n/a	MT015767
AT.p02.B04	*O. annulipes*	MT001327	Microsporidium sp. PL01	n/a	MT015768
AT.p02.C04	*O. annulipes*	MT001328	Microsporidium sp. PL01	n/a	MT015769
AT.p02.D04	*O. cantans*	MT001329	Microsporidium sp. PL01	n/a	MT015770
AT.p02.F04	*O. annulipes*	MT001330	Microsporidium sp. PL01	n/a	MT015771
AT.p02.G04	*C. richiardii*	MT001331	Microsporidium sp. PL01	n/a	MT015772
AT.p02.H04	*O. cantans*	MT001332	Microsporidium sp. PL01	n/a	MT015773
AT.p02.A05	*A. vexans*	MT001333	Microsporidium sp. PL01	MT015712	MT015774
AT.p02.D05	*C. richiardii*	MT075549	*Gregarina* sp.	n/a	MT015899
AT.p02.F05	*O. cantans*	MT001334	*Amblyospora* sp.; Microsporidium sp. PL01	MT015713	MT015749; MT015775
AT.p02.G05	*O. annulipes*	MT001335	Microsporidium sp. PL01	MT015714	MT015776
AT.p02.H05	*A. cinereus*	MT001336	Microsporidium sp. PL01	MT015715	MT015777
AT.p02.B06	*O. annulipes*	MT001337	Microsporidium sp. PL01	MT015716	MT015778
AT.p02.C06	*O. cantans*	MT001338	Microsporidium sp. PL01; *N. ceranae*	n/a	MT015779; MT015862
AT.p02.D06	*O. cantans*	MT001339	*Nosema chrysorrhoeae/portugal*; *Nosema* sp. CHW-2007a	n/a	MT015878; MT015891
AT.p02.E06	*O. annulipes*	MT001340	Microsporidium sp. PL01; *Nosema chrysorrhoeae/portugal*; *Nosema sp.* CHW-2007a	n/a	MT015780; MT015879; MT015892
AT.p02.F06	*O. punctor*	MT001341	Microsporidium sp. PL01	n/a	MT015781
AT.p02.H06	*O. cantans*	MT001342	*Nosema chrysorrhoeae/portugal*	n/a	MT015880
AT.p02.A07	*C. richiardii*	MT001343	Microsporidium sp. PL01	MT015717	MT015782
AT.p02.B07	*O. cantans*	MT001344	Microsporidium sp. PL01	n/a	MT015783
AT.p02.C07	*O. cantans*	MT001345	*E. hellem*; Microsporidium sp. PL01	n/a	MT015751; MT015784
AT.p02.E07	*O. cantans*	MT001346	Microsporidium sp. PL01	MT015718	MT015785
AT.p02.F07	*O. annulipes*	MT001347	Microsporidium sp. PL01	MT015719	MT015786
AT.p02.G07	*O. cantans*	MT001348	Microsporidium sp. PL01	MT015720	MT015787
AT.p02.H07	*C. richiardii*	MT001349	*Nosema chrysorrhoeae/portugal*	n/a	MT015881
AT.p02.C08	*O. cantans*	MT001350	Microsporidium sp. PL01	MT015721	MT015788
AT.p02.H08	*C. richiardii*	MT001351	Microsporidium sp. PL01	n/a	MT015789

Sample ID	Mosquito species	GenBank no.	OTU	GenBank no.	
		COI		V1 - V3 ssu rDNA	V5 ssu rDNA
AT.p02.D09	*O. cantans*	MT001352	Microsporidium sp. PL01	n/a	MT015790
AT.p02.F09	*O. cantans*	MT001353	*N. ceranae*; *Nosema chrysorrhoeae/portugal*	n/a	MT015863; MT015882
AT.p02.G09	*O. cantans*	MT001354	*Nosema chrysorrhoeae/portugal*	n/a	MT015883
AT.p02.H09	*C. richiardii*	MT001355	Microsporidium sp. PL01; *Nosema chrysorrhoeae/portugal*; *Nosema* sp. CHW-2007a	n/a	MT015791; MT015884; MT015893
AT.p02.A10	*O. cantans*	MT001356	Microsporidium sp. PL01	n/a	MT015792
AT.p02.C10	*O. annulipes*	MT001357	Microsporidium sp. PL01	MT015722	MT015793
AT.p02.E10	*O. cantans*	MT001358	Microsporidium sp. PL01	MT015723	MT015794
AT.p02.F10	*O. cantans*	MT001359	Microsporidium sp. PL01	MT015724	MT015795
AT.p02.H10	*O. cantans*	MT001360	Microsporidium sp. PL01	MT015725	MT015796
AT.p02.A11	*O. cantans*	MT001361	Microsporidium sp. PL01	MT015726	MT015797
AT.p02.B11	*O. annulipes*	MT001362	Microsporidium sp. PL01	MT015727	MT015798
AT.p02.D11	*O. cantans*	MT001363	*Nosema chrysorrhoeae/portugal*	n/a	MT015885
AT.p02.G11	*O. cantans*	MT001364	Microsporidium sp. PL01	n/a	MT015799
AT.p02.H11	*O. annulipes*	MT001365	Microsporidium sp. PL01	n/a	MT015800
AT.p02.A12	*O. annulipes*	MT001366	Microsporidium sp. PL01; *Nosema chrysorrhoeae/portugal*	n/a	MT015801; MT015886
AT.p02.B12	*C. richiardii*	MT075550	Uncultured fungus	n/a	MT015900
AT.p02.D12	*O. annulipes*	MT001367	*E. artemiae*; Microsporidium sp. PL01	n/a	MT015753; MT015802
AT.p02.E12	*O. cantans*	MT001368	*Nosema chrysorrhoeae/portugal*	n/a	MT015887
AT.p02.F12	*A. vexans*	MT001369	Microsporidium sp. PL01	n/a	MT015803
AT.p02.G12	*O. cantans*	MT001370	Microsporidium sp. PL01	MT015728	MT015804
AT.p02.H12	*O. annulipes*	MT001371	Microsporidium sp. PL01	n/a	MT015805
AT.p03.A01	*O. annulipes*	MT001372	Microsporidium sp. PL01	n/a	MT015806
AT.p03.B01	*O. punctor*	MT001373	Microsporidium sp. PL01	MT015729	MT015807
AT.p03.C01	*A. cinereus*	MT001374	Microsporidium sp. PL01	n/a	MT015808
AT.p03.D01	*O. annulipes*	MT001375	Microsporidium sp. PL01	MT015730	MT015809
AT.p03.E01	*O. cantans*	MT001376	Microsporidium sp. PL01	n/a	MT015810
AT.p03.F01	*O. cantans*	MT001377	*Nosema chrysorrhoeae/portugal*	n/a	MT015888
AT.p03.G01	*O. annulipes*	MT001378	Microsporidium sp. PL01	n/a	MT015811

Sample ID	Mosquito species	GenBank no.	OTU	GenBank no.	
		COI		V1 - V3 ssu rDNA	V5 ssu rDNA
AT.p03.H01	*O. annulipes*	MT001379	Microsporidium sp. PL01	MT015731	MT015812
AT.p03.B02	*O. annulipes*	MT001380	Microsporidium sp. PL01	n/a	MT015813
AT.p03.C02	*O. annulipes*	MT001381	Microsporidium sp. PL01	MT015732	MT015814
AT.p03.D02	*O. cantans*	MT001382	Microsporidium sp. PL01	n/a	MT015815
AT.p03.E02	*O. annulipes*	MT001383	Microsporidium sp. PL01	MT015733	MT015816
AT.p03.F02	*O. cantans*	MT001384	Microsporidium sp. PL01	n/a	MT015817
AT.p03.G02	*O. cantans*	MT001385	Microsporidium sp. PL01	n/a	MT015818
AT.p03.H02	*A. vexans*	MT001386	*Nosema chrysorrhoeae/portugal*	n/a	MT015889
AT.p03.A03	*O. cantans*	MT001387	Microsporidium sp. PL01	n/a	MT015819
AT.p03.B03	*A. vexans*	MT001388	Microsporidium sp. PL01	n/a	MT015820
AT.p03.C03	*O. punctor*	MT001389	Microsporidium sp. PL01; *N. adaliae*	n/a	MT015821; MT015859
AT.p03.D03	*O. cantans*	MT001390	Microsporidium sp. PL01	n/a	MT015822
AT.p03.E03	*A. cinereus*	MT001391	Microsporidium sp. PL01	n/a	MT015823
AT.p03.F03	*A. cinereus*	MT001392	Microsporidium sp. PL01	MT015734	MT015824
AT.p03.G03	*A. vexans*	MT001393	Microsporidium sp. PL01; Uncultured fungus	n/a	MT015825; MT015901
AT.p03.H03	*A. vexans*	MT001394	Microsporidium sp. PL01	n/a	MT015826
AT.p03.A04	*C. richiardii*	MT001395	Microsporidium sp. PL01	MT015735	MT015827
AT.p03.B04	*A. cinereus*	MT001396	Microsporidium sp. PL01	n/a	MT015828
AT.p03.D04	*O. annulipes*	MT001397	Microsporidium sp. PL01	MT015736	MT015829
AT.p03.F04	*C. richiardii*	MT001398	Microsporidium sp. PL01; *N. pieriae*	MT015737	MT015830; MT015866
AT.p03.G04	*O. punctor*	MT001399	Microsporidium sp. PL01	n/a	MT015831
AT.p03.A05	*O. punctor*	MT001400	Microsporidium sp. PL01	n/a	MT015832
AT.p03.B05	*O. sticticus*	MT001401	Microsporidium sp. PL01	n/a	MT015833
AT.p03.C05	*O. sticticus*	MT001402	Microsporidium sp. PL01	n/a	MT015834
AT.p03.D05	*O. sticticus*	MT001403	Microsporidium sp. PL01; *Nosema* sp. CHW-2007a	n/a	MT015835; MT015894
AT.p03.E05	*O. cantans*	MT001404	*E. hellem*; Microsporidium sp. PL01	MT015738	MT015752; MT015836
AT.p03.F05	*A. vexans*	MT001405	Microsporidium sp. PL01	MT015739	MT015837
AT.p03.G05	*O. punctor*	MT001406	Microsporidium sp. PL01	n/a	MT015838
AT.p03.H05	*O. punctor*	MT001407	Microsporidium sp. PL01	n/a	MT015839

Sample ID	Mosquito species	GenBank no.	OTU	GenBank no.	
		COI		V1 - V3 ssu rDNA	V5 ssu rDNA
AT.p03.A06	*A. cinereus*	MT001408	Microsporidium sp. PL01	MT015740	MT015840
AT.p03.B06	*O. annulipes*	MT001409	Microsporidium sp. PL01; *N. adaliae*; *Nosema* sp. CHW-2007a	MT015741	MT015841; MT015860; MT015895
AT.p03.C06	*O. sticticus*	MT001410	Microsporidium sp. PL01	n/a	MT015842
AT.p03.D06	*C. richiardii*	MT001411	Microsporidium sp. PL01	MT015742	MT015843
AT.p03.F06	*O. cantans*	MT001412	Microsporidium sp. PL01	n/a	MT015844
AT.p03.G06	*O. punctor*	MT001413	Microsporidium sp. PL01	n/a	MT015845
AT.p03.H06	*A. cinereus*	MT001414	Microsporidium sp. PL01	n/a	MT015846
AT.p03.A07	*O. annulipes*	MT001415	Microsporidium sp. PL01	n/a	MT015847
AT.p03.B07	*A. vexans*	MT001416	Microsporidium sp. PL01	n/a	MT015848
AT.p03.C07	*A. vexans*	MT001417	Microsporidium sp. PL01	n/a	MT015849
AT.p03.D07	*A. vexans*	MT001418	Microsporidium sp. PL01; *N. thomsoni*; *Nosema* sp. CHW-2007a	n/a	MT015850; MT015897; MT015896
AT.p03.E07	*O. sticticus*	MT001419	*Amblyospora* sp.; *Amblyospora salinaria*	n/a	MT015750; MT015747
AT.p03.G07	*A. cinereus*	MT001420	Microsporidium sp. PL01	n/a	MT015851
AT.p03.H07	*A. cinereus*	MT001421	Microsporidium sp. PL01	MT015743	MT015852
AT.p03.A08	*A. cinereus*	MT001422	Microsporidium sp. PL01	MT015744	MT015853
AT.p03.B08	*O. annulipes*	MT001423	Microsporidium sp. PL01	n/a	MT015854
AT.p03.C08	*O. annulipes*	MT001424	Microsporidium sp. PL01	MT015745	MT015855
AT.p03.D08	*A. cinereus*	MT001425	Microsporidium sp. PL01; *N. adaliae*	n/a	MT015856; MT015861
AT.p03.E08	*O. annulipes*	MT001426	Microsporidium sp. PL01	MT015746	MT015857
AT.p03.F08	*O. annulipes*	MT001427	Microsporidium sp. PL01	n/a	MT015858

Table S2. DNA sequences used in phylogenetic analysis.

Group	Clade	Species	GenBank no.
Ascomycota		*Graphis scripta*	AF038878.1
Ascomycota		*Meliniomyces variabilis*	AY838789.1
Ascomycota		*Nigrospora oryzae*	AB220234.1
Ascomycota		*Penicillium commune*	AF236103.1
Ascomycota		*Pseudosplanchnonema phorcioides*	KY984434.1
Basidiomycota		*Boletus edulis*	DQ534675.1
Basidiomycota		*Cryptococcus surugaensis*	AB100440.1
Basidiomycota		*Entyloma ficariae*	KP322949.1
Basidiomycota		*Leucosporidium fragarium*	KJ708437.1
Basidiomycota		*Malassezia equina*	KF706454.1
Basidiomycota		*Pachnocybe ferruginea*	DQ241440.1
Basidiomycota		*Sclerotium hydrophilum*	KC354147.1
Blastocladiomycota		*Allomyces arbuscula*	AY552524.1
Blastocladiomycota		*Allomyces javanicus*	JN941233.1
Blastocladiomycota		*Blastocladiella emersonii*	M54937.1
Blastocladiomycota		*Coelomomyces lativittatus*	HQ888713.1
Blastocladiomycota		*Coelomomyces quadrangulatus*	HQ888711.1
Blastocladiomycota		*Microallomyces dendroideus*	AY635840.1
Choanoflagellata		*Diaphanoeca grandis*	AF084234.1
Choanoflagellata		*Didymoeca costata*	EU011923.1
Choanoflagellata		*Helgoeca nana*	KT757452.1
Choanoflagellata		*Monosiga brevicollis*	AF100940.1
Choanoflagellata		*Savillea micropora*	EU011928.1
Chytridiomycota		*Lobulomyces angularis*	AF164253.2
Chytridiomycota		*Polychytrium aggregatum*	AY601711.1
Chytridiomycota		*Spizellomyces acuminatus*	M59759.1
Chytridiomycota		*Zygophlyctis asterionellae*	LC176289.1
Chytridopsida		*Chytridiopsis typographi*	MH728789.1
Classical microsporidia	Clade I	*Amblyospora californica*	U68473.1
Classical microsporidia	Clade I	*Amblyospora connecticus*	AF025685.1
Classical microsporidia	Clade I	*Amblyospora salinaria*	AY326270.1
Classical microsporidia	Clade I	*Amblyospora* sp.	AY090055.1
Classical microsporidia	Clade I	*Culicosporella lunata*	AF027683.1
Classical microsporidia	Clade I	*Gurleya daphniae*	AF439320.1
Classical microsporidia	Clade I	*Hazardia milleri*	AY090067.1
Classical microsporidia	Clade I	*Hyalinocysta chapmani*	AF483837.1
Classical microsporidia	Clade I	*Marssoniella elegans*	AY090041.1
Classical microsporidia	Clade I	*Octosporea muscaedomesticae*	FN794114.1
Classical microsporidia	Clade I	*Parathelohania anophelis*	AF027682.1
Classical microsporidia	Clade II	*Antonospora psocopterae*	FJ865222.1
Classical microsporidia	Clade II	*Antonospora scoticae*	AF024655.1
Classical microsporidia	Clade II	*Nematocida ciargi*	KX360152.1
Classical microsporidia	Clade II	*Nematocida homosporus*	KX360153.1
Classical microsporidia	Clade II	*Nematocida minor*	KX360151.1
Classical microsporidia	Clade II	*Nematocida parisii*	XR_001214623.1
Classical microsporidia	Clade II	*Ovavesicula popilliae*	EF564602.1
Classical microsporidia	Clade II	*Paranosema grylli*	AY305325.1
Classical microsporidia	Clade II	*Paranosema locustae*	AY305324.1

Group	Clade	Species	GenBank no.
Classical microsporidia	Clade III	*Anncaliia algerae*	HM216911.1
Classical microsporidia	Clade III	*Bacillidium vesiculoformis*	AJ581995.1
Classical microsporidia	Clade III	*Bryonosema plumatellae*	AF484691.1
Classical microsporidia	Clade III	*Janacekia debaisieuxi*	AJ252950.1
Classical microsporidia	Clade III	*Kneallhazia carolinensae*	GU173849.1
Classical microsporidia	Clade III	*Systenostrema alba*	AY953292.2
Classical microsporidia	Clade III	*Trichonosema pectinatellae*	AF484695.1
Classical microsporidia	Clade III	*Tubulinosema kingi*	DQ019419.1
Classical microsporidia	Clade IV	*Cystosporogenes legeri*	AY233131.2
Classical microsporidia	Clade IV	*Cystosporogenes operophterae*	AJ302320.1
Classical microsporidia	Clade IV	*Encephalitozoon cuniculi*	AJ005581.1
Classical microsporidia	Clade IV	*Encephalitozoon hellem*	AF272836.1
Classical microsporidia	Clade IV	*Encephalitozoon intestinalis*	XR_002670132.1
Classical microsporidia	Clade IV	*Endoreticulatus bombycis*	AY009115.1
Classical microsporidia	Clade IV	*Endoreticulatus schubergi*	L39109.1
Classical microsporidia	Clade IV	*Enterocytospora artemiae*	JX915761.1
Classical microsporidia	Clade IV	*Enterocytozoon bieneusi*	L07123.1
Classical microsporidia	Clade IV	*Enterocytozoon hepatopenaei*	KX981865.1
Classical microsporidia	Clade IV	*Glugoides intestinalis*	AF394525.1
Classical microsporidia	Clade IV	*Heterovesicula cowani*	EU275200.1
Classical microsporidia	Clade IV	*Liebermannia dichroplusae*	EF016249.1
Classical microsporidia	Clade IV	Microsporidium sp.1199	FN610845.1
Classical microsporidia	Clade IV	*Mrazekia macrocyclopis*	FJ914315.1
Classical microsporidia	Clade IV	*Nosema adaliae*	KC412706.1
Classical microsporidia	Clade IV	*Nosema apis*	U97150.1
Classical microsporidia	Clade IV	*Nosema bombycis*	AY259631.1
Classical microsporidia	Clade IV	*Nosema ceranae*	MH356535
Classical microsporidia	Clade IV	*Nosema maddoxi*	KY783604.1
Classical microsporidia	Clade IV	*Nosema pieriae*	JX268035.1
Classical microsporidia	Clade IV	*Nosema portugal*	AF033316.1
Classical microsporidia	Clade IV	*Nosema* sp. CHW-2007a	EF585399.1
Classical microsporidia	Clade IV	*Nosema thomsoni*	KC596023.1
Classical microsporidia	Clade IV	*Nucleospora salmonis*	U78176.1
Classical microsporidia	Clade IV	*Ordospora colligata*	AF394529.1
Classical microsporidia	Clade IV	*Orthosomella operophterae*	AJ302316.1
Classical microsporidia	Clade IV	*Paranucleospora theridion*	FJ594990.1
Classical microsporidia	Clade IV	*Vairimorpha necatrix*	DQ996241.1
Classical microsporidia	Clade IV	*Vittaforma corneae*	U11046.1
Classical microsporidia	Clade V	*Ameson michaelis*	L15741.1
Classical microsporidia	Clade V	*Dictyocoela duebenum*	MG773213.1
Classical microsporidia	Clade V	*Kabatana takedai*	AF356222.1
Classical microsporidia	Clade V	*Loma acerinae*	AJ252951.1
Classical microsporidia	Clade V	*Loma salmonae*	HM626203.1
Classical microsporidia	Clade V	*Myospora metanephrops*	HM140491.1
Classical microsporidia	Clade V	*Myosporidium merluccius*	AY530532.1
Classical microsporidia	Clade V	*Pleistophora ehrenbaumi*	AF044392.1
Classical microsporidia	Clade V	*Pleistophora finisterrensis*	AF044393.1
Classical microsporidia	Clade V	*Pleistophora hippoglossoideos*	AF044388.1
Classical microsporidia	Clade V	*Pleistophora hyphessobryconis*	KM458272.1
Classical microsporidia	Clade V	*Pleistophora mulleri*	EF119339.1
Classical microsporidia	Clade V	*Pleistophora ovariae*	AJ252955.1

Group	Clade	Species	GenBank no.
Classical microsporidia	Clade V	*Pleistophora typicalis*	AF044387.1
Classical microsporidia	Clade V	*Spraguea lophii*	AF033197.2
Classical microsporidia	Clade V	*Vavraia culicis*	XR_552272.1
Glomeromycota		*Claroideoglomus lamellosum*	AJ276083.3
Glomeromycota		*Dentiscutata colliculosa*	GQ376067.1
Glomeromycota		*Geosiphon pyriformis*	AM183923.1
Glomeromycota		*Gigaspora margarita*	AJ852604.1
Glomeromycota		*Glomus intraradices*	FJ009592.1
Metchnikovellida		*Amphiacantha* sp. ex Lecudina cf. Elongata	KX214676.1
Metchnikovellida		*Amphiacantha* sp. ex Lecudina cf. Longissima	KX214677.1
Metchnikovellida		*Amphiamblys* sp. WSBS2006	KX214672.1
Metchnikovellida		*Amphiamblys* sp. WSBS2011	KX214674.1
Nucleariidae		*Nuclearia delicatula*	AF349563.1
Nucleariidae		*Nuclearia moebiusi*	AF349565.1
Nucleariidae		*Nuclearia pattersoni*	AY364635.1
Nucleariidae		*Nuclearia simplex*	AF349566.1
Nucleariidae		*Nuclearia thermophila*	LN875109.1
Rozellomycota		*Nucleophaga amoebae*	JQ288099.1
Rozellomycota		*Nucleophaga terricolae*	KX017226.1
Rozellomycota		*Paramicrosporidium saccamoebae*	JQ796369.1
Rozellomycota		*Paramicrosporidium vannellae*	JQ796368.1
Rozellomycota		*Rozella allomycis*	NG_017174.1
Rozellomycota		*Rozella rhizoclosmatii*	KX354829.1

Table S3. Pearson's correlation matrix of coefficient values. Bold values mean statistically significant correlations. *A. s.* – *A. salinaria*, *A.* sp. – *Amblyospora* sp., *E. h.* – *E. hellem*, *E. a.* – *E. artemiae*, M. sp. PL01 – Microsporidium sp. PL01, *N. a* – *N. adaliae*, *N. c.* – *N. ceranae*, *N. p.* – *N. pieriae*, *N. t.* – *N. thomsoni*, *N. ch./p.* – *Nosema* chrysorrhoeae and/or portugal, *N.* sp. CHW-2007a – *Nosema* CHW-2007a

**Microsporidium**	***E. h.***	***N. a.***	***N. p.***	***N. ch./p.***	***N. t.***	***N. c.***	**M. sp. PL01**	***N.* sp. CHW-2007a**	***A.* sp.**	***A. s.***
N. a.	-0.0093									
N. p.	-0.0084	-0.0115								
N. ch./p.	-0.007	-0.0097	0.0205							
N. t.	-0.0058	-0.008	-0.0072	-0.006						
N. c.	-0.0078	-0.0108	-0.0097	-0.0025	-0.0067					
**M. sp. PL01**	-0.0052	-0.0086	-0.0087	-0.007	0.0241	-0.0081				
***N.* sp. CHW-2007a**	-0.0101	0.0252	-0.0125	**0.9043**	0.1057	-0.0118	-0.01			
***A.* sp.**	-0.0093	-0.0128	-0.0115	-0.0097	-0.008	-0.0108	-0.0097	-0.014		
A. s.	-0.0056	-0.0078	-0.007	-0.0059	-0.0048	-0.0066	-0.0059	-0.0085	**0.512**	
E. a.	-0.0056	-0.0078	-0.007	-0.0059	-0.0048	-0.0066	-0.0059	-0.0085	-0.0078	-0.0047

Table S4. A comparison of metabarcoding and standard approach based on amplification and direct sequencing of V1-V3 ssu rDNA. *A. s.* – *A. salinaria*, *A*. sp. – *Amblyospora* sp. 1, *E. h.* – *E. hellem*, *E. a.* – *E. artemiae*, M. sp. PL01 – Microsporidium sp. PL01, *N. a* – *N. adaliae*, *N. c*. – *N. ceranae*, *N. p*. – *N. pieriae*, *N. t*. – *N. thomsoni*, *N. ch./p*. – *Nosema chrysorrhoeae and/or portugal*, *N*. sp. CHW-2007a – *Nosema* sp. CHW-2007a

**Mosquito species**	**Sample ID**	**Number of sequence reads in NGS data for the detected microsporidian species**											**Sanger sequencing**
		A. s.	***A. sp.***	***E. h.***	***E. a.***	**M. sp. PL01**	***N. a.***	***N. c.***	***N. p.***	***N. t.***	***N. ch./p.***	***N.* sp. CHW-2007a**	
O. sticticus	AT.p01.G05					10							no PCR product
O. sticticus	AT.p01.A06					1528							Microsporidium sp. PL01
A. vexans	AT.p01.B06											16	no PCR product
O. cantans	AT.p01.H06					18							no PCR product
O. sticticus	AT.p01.F07					16382							Microsporidium sp. PL01
O. annulipes	AT.p01.G07					19							no PCR product
O. annulipes	AT.p01.A08					16							no PCR product
O. cantans	AT.p01.B08										10		no PCR product
O. annulipes	AT.p01.C08								118		100		no PCR product
O. annulipes	AT.p01.D08								20		16		no PCR product
O. cantans	AT.p01.E08					10							no PCR product
O. cantans	AT.p01.H08										36		no PCR product
O. cantans	AT.p01.F09					2188							Microsporidium sp. PL01
O. cantans	AT.p01.G10										26		no PCR product
A. vexans	AT.p01.H10					38					584		no PCR product
O. cantans	AT.p01.G11										14		no PCR product
O. cantans	AT.p01.G12										14		no PCR product
O. annulipes	AT.p02.F01		32										*Amblyospora* sp.
O. cantans	AT.p02.G01										85		no PCR product
O. cantans	AT.p02.C02										11		no PCR product
O. annulipes	AT.p02.B03					18							no PCR product
O. cantans	AT.p02.C03										25		no PCR product
C. richiardii	AT.p02.E03					317							no PCR product
O. annulipes	AT.p02.F03					3414							Microsporidium sp. PL01
A. cinereus	AT.p02.H03					16							no PCR product
O. cantans	AT.p02.A04					124							no PCR product
O. annulipes	AT.p02.B04					306							no PCR product

Mosquito species	Sample ID	Number of sequence reads in NGS data for the detected microsporidian species											Sanger sequencing
O. annulipes	AT.p02.C04					134							no PCR product
O. cantans	AT.p02.D04					288							no PCR product
O. annulipes	AT.p02.F04					141							no PCR product
C. richiardii	AT.p02.G04					150							no PCR product
O. cantans	AT.p02.H04					18							no PCR product
A. vexans	AT.p02.A05					10439							Microsporidium sp. PL01
O. cantans	AT.p02.F05		14			426							Microsporidium sp. PL01
O. annulipes	AT.p02.G05					1413							Microsporidium sp. PL01
A. cinereus	AT.p02.H05					522							Microsporidium sp. PL01
O. annulipes	AT.p02.B06					35							Microsporidium sp. PL01
O. cantans	AT.p02.C06					544		16					no PCR product
O. cantans	AT.p02.D06										25	21	no PCR product
O. annulipes	AT.p02.E06					1283					3080	73	unreadable chromatogram
O. punctor	AT.p02.F06					33							no PCR product
O. cantans	AT.p02.H06										12		no PCR product
C. richiardii	AT.p02.A07					192765							Microsporidium sp. PL01
O. cantans	AT.p02.B07					120							no PCR product
O. cantans	AT.p02.C07			31		6766							no PCR product
O. cantans	AT.p02.E07					368							Microsporidium sp. PL01
O. annulipes	AT.p02.F07					286							Microsporidium sp. PL01
O. cantans	AT.p02.G07					865							Microsporidium sp. PL01
C. richiardii	AT.p02.H07										11		no PCR product
O. cantans	AT.p02.C08					116							Microsporidium sp. PL01
C. richiardii	AT.p02.H08					19							no PCR product
O. cantans	AT.p02.D09					11							no PCR product
O. cantans	AT.p02.F09							19			21		no PCR product
O. cantans	AT.p02.G09										98		no PCR product
C. richiardii	AT.p02.H09					22					19	22	no PCR product
O. cantans	AT.p02.A10					140							no PCR product
O. annulipes	AT.p02.C10					51306							Microsporidium sp. PL01
O. cantans	AT.p02.E10					454							Microsporidium sp. PL01

Mosquito species	Sample ID	Number of sequence reads in NGS data for the detected microsporidian species											Sanger sequencing
O. cantans	AT.p02.F10					2857							Microsporidium sp. PL01
O. cantans	AT.p02.H10					753							Microsporidium sp. PL01
O. cantans	AT.p02.A11					1615							Microsporidium sp. PL01
O. annulipes	AT.p02.B11					26152							Microsporidium sp. PL01
O. cantans	AT.p02.D11										11		no PCR product
O. cantans	AT.p02.G11					76							no PCR product
O. annulipes	AT.p02.H11					19							no PCR product
O. annulipes	AT.p02.A12					15					15		no PCR product
O. annulipes	AT.p02.D12				30	19							no PCR product
O. cantans	AT.p02.E12										12		no PCR product
A. vexans	AT.p02.F12					237							no PCR product
O. cantans	AT.p02.G12					91							Microsporidium sp. PL01
O. annulipes	AT.p02.H12					37							no PCR product
O. annulipes	AT.p03.A01					116							no PCR product
O. punctor	AT.p03.B01					645							Microsporidium sp. PL01
A. cinereus	AT.p03.C01					89							no PCR product
O. annulipes	AT.p03.D01					505							Microsporidium sp. PL01
O. cantans	AT.p03.E01					42							no PCR product
O. cantans	AT.p03.F01										17		no PCR product
O. annulipes	AT.p03.G01					211							no PCR product
O. annulipes	AT.p03.H01					791							Microsporidium sp. PL01
O. annulipes	AT.p03.B02					10							no PCR product
O. annulipes	AT.p03.C02					26845							Microsporidium sp. PL01
O. cantans	AT.p03.D02					10							no PCR product
O. annulipes	AT.p03.E02					2600							Microsporidium sp. PL01
O. cantans	AT.p03.F02					31							no PCR product
O. cantans	AT.p03.G02					22							no PCR product
A. vexans	AT.p03.H02										39		no PCR product
O. cantans	AT.p03.A03					28							no PCR product
A. vexans	AT.p03.B03					51							no PCR product
O. punctor	AT.p03.C03					18	56						no PCR product

Mosquito species	Sample ID	Number of sequence reads in NGS data for the detected microsporidian species											Sanger sequencing
O. cantans	AT.p03.D03					41							no PCR product
A. cinereus	AT.p03.E03					251							no PCR product
A. cinereus	AT.p03.F03					23760							Microsporidium sp. PL01
A. vexans	AT.p03.G03					23							no PCR product
A. vexans	AT.p03.H03					54							no PCR product
C. richiardii	AT.p03.A04					591							Microsporidium sp. PL01
A. cinereus	AT.p03.B04					209							no PCR product
O. annulipes	AT.p03.D04					8795							Microsporidium sp. PL01
C. richiardii	AT.p03.F04					372			59				Microsporidium sp. PL01
O. punctor	AT.p03.G04					414							no PCR product
O. punctor	AT.p03.A05					111							no PCR product
O. sticticus	AT.p03.B05					90							no PCR product
O. sticticus	AT.p03.C05					96							no PCR product
O. sticticus	AT.p03.D05					22						16	no PCR product
O. cantans	AT.p03.E05			17		503							Microsporidium sp. PL01
A. vexans	AT.p03.F05					659							Microsporidium sp. PL01
O. punctor	AT.p03.G05					99							no PCR product
O. punctor	AT.p03.H05					59							no PCR product
A. cinereus	AT.p03.A06					390							Microsporidium sp. PL01
O. annulipes	AT.p03.B06					1647	118					15	Microsporidium sp. PL01
O. sticticus	AT.p03.C06					75							no PCR product
C. richiardii	AT.p03.D06					1153							Microsporidium sp. PL01
O. cantans	AT.p03.F06					18							no PCR product
O. punctor	AT.p03.G06					100							no PCR product
A. cinereus	AT.p03.H06					11							no PCR product
O. annulipes	AT.p03.A07					82							no PCR product
A. vexans	AT.p03.B07					350							no PCR product
A. vexans	AT.p03.C07					186							no PCR product
A. vexans	AT.p03.D07					8000				2948		19	unreadable chromatogram
O. sticticus	AT.p03.E07	24	21										no PCR product
A. cinereus	AT.p03.G07					430							no PCR product

Mosquito species	Sample ID	Number of sequence reads in NGS data for the detected microsporidian species											Sanger sequencing
A. cinereus	AT.p03.H07					538226							Microsporidium sp. PL01
A. cinereus	AT.p03.A08					684							Microsporidium sp. PL01
O. annulipes	AT.p03.B08					129							no PCR product
O. annulipes	AT.p03.C08					199							Microsporidium sp. PL01
A. cinereus	AT.p03.D08					3924	141						no PCR product
O. annulipes	AT.p03.E08					104							Microsporidium sp. PL01
O. annulipes	AT.p03.F08					172							no PCR product
